# Evolution of the adaptogenic concept from traditional use to medical systems: Pharmacology of stress‐ and aging‐related diseases

**DOI:** 10.1002/med.21743

**Published:** 2020-10-25

**Authors:** Alexander G. Panossian, Thomas Efferth, Alexander N. Shikov, Olga N. Pozharitskaya, Kenny Kuchta, Pulok K. Mukherjee, Subhadip Banerjee, Michael Heinrich, Wanying Wu, De‐an Guo, Hildebert Wagner

**Affiliations:** ^1^ Phytomed AB Vaxtorp Sweden; ^2^ Department of Pharmaceutical Biology, Institute of Pharmacy and Biochemistry Johannes Gutenberg University Mainz Germany; ^3^ Department of technology of dosage forms Saint‐Petersburg State Chemical‐Pharmaceutical University St. Petersburg Russia; ^4^ Department of Biotechnology Murmansk Marine Biological Institute of the Kola Science Center of the Russian Academy of Sciences (MMBI KSC RAS) Murmansk Russia; ^5^ Department of Far Eastern Medicine, Clinic for Gastroenterology and Gastrointestinal Oncology University Medical Center Göttingen Göttingen Germany; ^6^ Department of Pharmaceutical Technology, School of Natural Product Studies Jadavpur University Kolkata India; ^7^ Research Cluster Biodiversity and Medicines, UCL School of Pharmacy, Centre for Pharmacognosy and Phytotherapy University of London London UK; ^8^ Shanghai Research Center for TCM Modernization, Shanghai Institute of Materia Medica Chinese Academy of Sciences Shanghai China; ^9^ Department of Pharmacy, Center for Pharma Research Ludwig‐Maximilians‐Universität München Munich Germany

**Keywords:** adaptogen, aging, ethnopharmacology, network pharmacology, stress

## Abstract

Adaptogens comprise a category of herbal medicinal and nutritional products promoting adaptability, resilience, and survival of living organisms in stress. The aim of this review was to summarize the growing knowledge about common adaptogenic plants used in various traditional medical systems (TMS) and conventional medicine and to provide a modern rationale for their use in the treatment of stress‐induced and aging‐related disorders. Adaptogens have pharmacologically pleiotropic effects on the neuroendocrine‐immune system, which explain their traditional use for the treatment of a wide range of conditions. They exhibit a biphasic dose‐effect response: at low doses they function as mild stress‐mimetics, which activate the adaptive stress‐response signaling pathways to cope with severe stress. That is in line with their traditional use for preventing premature aging and to maintain good health and vitality. However, the potential of adaptogens remains poorly explored. Treatment of stress and aging‐related diseases require novel approaches. Some combinations of adaptogenic plants provide unique effects due to their synergistic interactions in organisms not obtainable by any ingredient independently. Further progress in this field needs to focus on discovering new combinations of adaptogens based on traditional medical concepts. Robust and rigorous approaches including network pharmacology and systems pharmacology could help in analyzing potential synergistic effects and, more broadly, future uses of adaptogens. In conclusion, the evolution of the adaptogenic concept has led back to basics of TMS and a new level of understanding of holistic approach. It provides a rationale for their use in stress‐induced and aging‐related diseases.

## 1 INTRODUCTION

Numerous systematic reviews, meta‐analyses of preclinical and clinical studies, and comprehensive assessment reports^1^, ^2^, ^3^, ^4^, ^5^, ^6^, ^7^, ^8^, ^9^, ^10^, ^11^, ^12^, ^13^, ^14^, ^15^, ^16^, ^17^ on the efficacy and safety of adaptogenic plants have been published in the last several decades. The aim of this review is to summarize our knowledge about common concept relating to adaptogenic plants used as officinal medical preparations in the USSR/Russian and in traditional Chinese medicine (TCM), Ayurveda, Kampo, and other traditional medical systems (TMS) and alternative medical systems, and to analyze how such preparations have been studied scientifically. This provides a basis for assessing the use of adaptogens in the treatment of stress‐induced and aging‐related disorders.

Adaptogens must be innocuous and cause minimal disorder in the physiological functions of an organism, and have nonspecific actions, that is, increase resistance to adverse influences of a wide range of factors with physical, chemical, and biological properties. In addition, they typically possess normalizing actions irrespective of the direction of the foregoing pathologic changes.

### 1.1 Evolution of the adaptogenic concept: From postulates to evidence‐based statements

The term adaptogens is currently widely used in alternative and complementary medicine, as well as in pharmacognosy, phytomedicine, and phytotherapy research.^5^ It was implemented in scientific lexicon in the middle of the 20th century in the Soviet Union with the aim of characterizing the physiological mechanisms of action of compounds and some medicinal plants that presumably increased the nonspecific resilience of organisms to harmful challenges. The definition of adaptogens is continuously updated (Table 1), incorporating the increasing body of scientific evidence related to understanding their pharmacological and molecular mechanisms of action.

**Table 1:** Definitions of adaptogens

Adaptogens are *medicinal substances* causing the “state of nonspecifically increased resistance” of the organism.^6^, ^7^
Only those preparations that meet the following requirements may be included in the group of adaptogens: (a) An adaptogen should be innocuous and cause minimal disorders in the physiological functions of an organism; (b) The action of an adaptogen should be nonspecific, i.e., it should increase resistance to adverse influences of a wide range of factors of physical, chemical and biological nature, (c) An adaptogen may possess normalizing action irrespective of the direction of the foregoing pathologic changes.^8^
The adaptogens are nontoxic *compounds* with polyvalent mechanisms of action and pharmacological effects related to adaptability and survival.^9^
Adaptogens are *substances*, which elicit in an organism a state of nonspecifically raised resistance, allowing them to counteract stressor signals and to adapt to exceptional strain.^10^
Adaptogens are *metabolic regulators*, which increase the ability of an organism to adapt to environmental factors and to avoid damage from such factors.^11^
Plant adaptogens are *agents*, which reduce damaging effects of various stressors due to reduction of the reactivity of host defense system. They adapt organism to stress and have curative effect in stress‐induced disorders.^12^
Adaptogenic *substances* have the capacity to normalize body functions and strengthen systems compromised by stress. They have a protective effect on health against a wide variety of environmental assaults and emotional conditions.^13^
Adaptogens comprise *a pharmacotherapeutic group of herbal preparations* used to: increase attention and endurance in fatigue and prevent/mitigate/reduce stress‐induced impairments and disorders related to neuro‐endocrine and immune systems.^14^
*Botanical* adaptogens are *plant extracts*, or specific constituents of plant extracts, which function to increase survival in animals and humans by stimulating their adaptability to stress by inducing adaptive responses.^15^
Adaptogens are *stress‐response modifiers* that increase an organism's nonspecific resistance to stress by increasing its ability to adapt and survive.^16^
Botanical adaptogens are *metabolic regulators* that increase survival by increasing adaptability in stress.^16^
Adaptogens are *natural compounds* or *plant extracts* that increase adaptability and survival of living organisms to stress.^17^
Adaptogen—any of various *natural substances* used in herbal medicine to normalize and regulate the systems of the body. https://www.dictionary.com/browse/adaptogen

Importantly, the term adaptogen is related to a physiological process—adaptation to environmental challenges, which is a multistep process including diverse mechanisms of extracellular and intracellular interactions. The renewed definition of adaptogens^16^, ^17^ is supported by the results of recent studies on the molecular mechanisms of action of adaptogens in a variety of regulatory systems from the cellular to entire organism levels.^11^, ^16^, ^17^, ^18^, ^19^, ^20^, ^21^, ^22^, ^23^, ^24^, ^25^, ^26^, ^27^, ^28^, ^29^, ^30^, ^31^, ^32^, ^33^, ^34^, ^35^, ^36^, ^37^, ^38^, ^39^, ^40^, ^41^, ^42^, ^43^, ^44^, ^45^, ^46^, ^47^, ^48^, ^49^, ^50^, ^51^, ^52^, ^53^, ^54^, ^55^, ^56^, ^57^, ^58^, ^59^, ^60^, ^61^, ^62^, ^63^

Similar to antioxidants and vitamins, adaptogens constitute a category of nutritional and herbal medicinal products essential for good health, adaptability, resilience, survival, and healthy aging. Regardless of the nature of the stimulus (stressor), an adaptogen increases adaptability, resilience, and survival by activating adaptive signaling pathways of cellular and organismal defence systems (stress system e.g., neuroendocrine‐immune complex). Furthermore, adaptogens trigger the generation of hormones (cortisol, corticotropin‐releasing hormone [CRH] and gonadotropin‐releasing hormones, urocortin, neuropeptide Y), playing key roles in metabolic regulation and homeostasis. Meanwhile, multitarget mechanisms of action and a wide range of pharmacological effects indicate their nonspecific pharmacological activity.

Therefore, adaptogens are most likely effective for the prevention and treatment of stress‐induced and adult‐onset disorders such as chronic fatigue, memory impairment, depression, anxiety, sleep disturbance, diabetes, heart disease and high blood pressure, chronic inflammation and autoimmune diseases, cold and flu, infections, skin diseases, liver diseases, and cancer. This can be achieved due to their ability to activate the innate defence system, increase resistance to stress, adapt organisms to stress, increase recovery of stress‐induced damages, provide energy to fight fatigue, reduce aging‐associated decline of the neuroendocrine‐immune system. Table 2 provides a summary of the general characteristics of adaptogens, which comprise a category of nutritional and herbal medicinal products.

**Table 2:** Summary of characteristics of adaptogens

*Definition*: Adaptogens are natural compounds or plant extracts that increase adaptability, resilience, and survival of organisms to stress.
*Chemical class*: Various, predominantly tetracyclic triterpenes, phenethyl‐and phenylpropanoids glycosides, stilbenes, lignans, etc.
*Pharmacological activity/health claims*: adaptogenic
*Mechanism of action*: Multitarget effects on neuroendocrine‐immune system including:
(i) Triggering of intracellular and extracellular adaptive signaling pathways that promote cell survival and organismal resilience in stress
(ii) Regulation of metabolism and homeostasis via effects on expression of stress hormones (corticotropin and gonadotropin‐releasing hormones, urocortin, cortisol, neuropeptide Y, heat shock proteins Hsp70) and their receptors.
*Indications for use*: Stress‐induced fatigue, mental and behavioral disorders, aging‐associated diseases.

## 2 BACKGROUND OF THE ADAPTOGENIC CONCEPT

### 2.1 Origin of the adaptogenic concept and use in officinal medicine of the USSR

The term adaptogen was introduced in 1958 by the Soviet toxicologist Lazarev, who applied it to the synthetic stimulant dibazol (2‐phenyl‐imidazol) assuming that adaptogens increase the nonspecific resistance of organisms under conditions of stress resulting in increased endurance, stamina, and performance.^6^ This assumption was based on the results of intensive studies of *Schisandra chinensis* in the USSR during World War II,^64^, ^65^, ^66^ with the goal of finding an alternative to stimulants used by the German and U.K. army to increase the attention and endurance of pilots.^67^ The aim was also to supply the Soviet Armed Forces and Military Industry (soldiers, pilots, sailors, and civilians engaged in the production of weapons and war materials) with easily available natural stimulants, presumably extracts from *S. chinensis* berry or seeds.^68^

The interest in *S. chinensis* (known as *limonnik = лимонник* in Russian) arose from ethnopharmacological investigations by. Komarov (1895) and Arsenyev (1903–1907) in far eastern Siberia and northern Manchuria. The berries and seeds were determined to have been used by Nanai hunters (natives of far eastern Siberia and Chinese Manchuria, also known as Goldis or Samagir) as a tonic to reduce thirst, hunger, and exhaustion and to improve night‐time vision.^69^

The first studies on the stimulating and tonic effects on *S. chinensis* were published in World War II‐era military journals.^64^, ^65^, ^66^ During the 1960s and 1970s, other Soviet scientists extended the research of adaptogens to “rejuvenating and invigorating” medicinal plants traditionally used in China, Korea, Japan, Siberia, and the far east of the USSR for a variety of pathological conditions including diseases and their symptoms such as hypodynamia, asthenia, shortness of breath, palpitation, insomnia, hemorrhage, impotence, and diabetes.^70^, ^71^, ^72^

The authors screened many plants assuming that “adaptogens must be safe and normalize body functions irrespective of the nature of stressors” and in 1967, some were incorporated into official medical practice in the USSR as central nervous system (CNS)‐stimulating medicinal products and as tonics to fight fatigue and general weakness during convalescence for infectious diseases, chemotherapy and psychiatric disorders, after surgery, poisoning, heart attacks, ischemia, chemotherapy, and psychiatric disorders (Table 3). *Rhodiola rosea* extract (Rhodiolae roseae rhizomatum et radicum extractum liquidum) is an example of an adaptogenic medicinal product used since 1975 in officinal medicine in the USSR/Russia. It is indicated for “decreased mental and physical capacities such as weakness, exhaustion, tiredness and loss of concentration, as well as during convalescence.” The extent of adaptogen research conducted in the USSR was enormous with more than 1000 pharmacological and clinical studies published in Russia until 1982.

**Table 3:** Adaptogenic plants used in officinal medicine in the USSR/Russia^a^

Name of plant	Products	Pharmacopoeia monograph
*Aralia elata (Miq.) Seem (A. mandshurica Rupr. et maxim.)*	Radices	FS.2.5.0058.18
	Tincture	FS 42‐1647‐93
	Dry extract in tablets	FS 42‐1755‐81
*Eleutherococcus senticosus (Rupr. & Maxim.) Maxim*.	Radices and rhizomes,	FS.2.5.0053.15
	Liquid extract	FS.3.4.0009.18
*Oplopanax elatus (Nakai) Nakai (Echinopanax elatum* Nakai)	Radices and rhizomes,	FS 42‐314‐72
	Tincture	FS 42‐1887‐82
*Panax ginseng C.A*. Meyer	Radices	FS.2.5.0013.15
	Tincture	FS 42‐1886‐82
*Rhaponticum carthamoides* (Willd).Iljin	Radices and rhizomes,	FS.2.5.0091.18
	Liquid extract	FS 42‐1995‐99
*Rhodiola rosea* L. (a synonym of *Sedum roseum* (L.) Scop.)	Radices and rhizomes,	FS.2.5.0036.15
	Liquid extract	FS.3.4.0008.18
*Schisandra chinensis* (Turcz.) Bail.	Fruits	FS.2.5.0081.18
	Seeds	FS.2.5.0082.18
	Tincture from seeds,	FS 42‐1822‐90
	Tincture from fruits,	VFS 42‐117‐72
	Oil from seeds in capsules	VFS 42‐3423‐99

^a^ The State Pharmacopoeia of the Russian Federation, 2018. http://femb.ru/femb/pharmacopea.php (Accessed date: March 15, 2020).

Most common extracts or compounds isolated from Siberian Ginseng (*Eleutherococcus senticosus*), Schisandra (*S. chinensis*), Ginseng (*Panax ginseng*), and Golden Root (*R. rosea*) have been studied. All adaptogenic plants and preparations from them have been clinically tested and approved before incorporation into official medical practice. The list of clinically approved true adaptogenic plants with related pharmacopeial monographs is presented in Table 3.

Regardless of the formal indication for use in officinal medicine as tonics, adaptogens were widely used in:

- sports medicine to promote quicker recovery after heavy exercise and overstraining,
- occupational medicine for protection against negative environmental factors, and
- geriatric medicine with the aim of promoting health by preventing and treating diseases and disabilities in older adults.

These areas of practical use of adaptogens were of socioeconomic importance in the USSR, a superpower where great achievements in space, military power, and sports have been the subjects of pride and special attention. Indeed, adaptogens were used in space medicine by Soviet cosmonauts during long missions on the MIR station,^73^, ^74^ as well as by sailors aboard ships; on submarines during long Arctic, Antarctic, or tropical expeditions; and by pilots and sportsmen in multiple stressful conditions such as hypoxia, irradiation, cold, and physical and mental overload. In addition, adaptogens termed “Kremlin Magic Pills” and “Elixir of Youth” that increase strength, stamina, and longevity were popular among elite elderly leaders of Communistic Party of the USSR, which governed the country for many years.

*In conclusion*, the concept of adaptogens can be traced back to their first definitions provided by the Soviet scientists Lazarev and Brekhman, and the introduction of herbal medicinal products as official medicaments and in the State pharmacopoeia of the USSR.

### 2.2 Ethnopharmacological background

Key points of the adaptogenic concept defined by Brekhman and Dardymov in 1969 are in line with basic principles of the TMS of China, Korea, Japan, India (Ayurveda), and Middle Asia (Yunani).

For instance, an assumption is that some adaptogens used in TCM, Kampo, and Ayurveda medicine (e.g., Ginseng, Ashwagandha, Andrographis, Bryony) must have normalizing effects, irrespective of the nature of the disease. Herbalists refer to adaptogens as restoratives, qi‐tonics, rasayanas, or rejuvenating herbs. Tonic herbs are classified as the highest and most sought‐after herbal remedies in many traditional systems of healing such as TCM and Ayurveda. Both traditional systems are based on holistic approaches to patients and treatment, suggesting that the patient is an individual and not a disease. Holistic medicine strives to consider the whole person, suggesting that one can only achieve optimal health by complex treatment of all imbalances (physical, emotional, or spiritual) induced by environmental factors. Consequently, multitarget therapy by herbal preparations have polyvalent actions on various mediators, effectors, and regulatory systems, presumably making it the most effective approach for the treatment of complex diseases.

Both TMS have a similar notion of “life vital energy” and activating the body and mind: the qi in TCM and the prana in Ayurveda. Similar notions exist in various cultures including the Greek *pneuma*, the Armenian *zorutyun* (զորություն), the Polynesian *mana*, the German *od*, and the Hebrew *ruah*. Prana is also referred to as life force, subtle, or bioplasmic energy. Below are brief descriptions of the ethnopharmacological roots of the adaptogenic concept.

#### 2.2.1 Traditional Chinese, Korean, and Japanese medicines

TCM is about 5000 years old, so billions of people in China (the world's biggest population with ~1.4 billion) have been treated with these herbal medicines/botanicals for centuries.

The core of the TCM concept is the yin‐yang theory consisting of two natural, complementary, and contradictory forces of opposite polarity that interact to form a dynamic system in which the entire is dual and better/superior than the collected parts. According to this philosophy, everything has both yin and yang features (for instance, shadow cannot exist without light), which are in dynamic equilibrium (balance); yin is negative/passive/dark/female/water, while yang is positive/active/bright/male/fire. Although yin is stronger, they are always in balance.

We can find many relevant examples of the yin‐yang balance when this concept is applied to the regulation of cellular and organismal homeostasis^75^ (e.g., cyclic adenosine monophosphate [cAMP] and cyclic guanosine monophosphate [c‐GMP], prostacyclin and thromboxane, sympathetic and parasympathetic nervous systems, testosterone, cortisol). For example, the testosterone/cortisol ratio is associated with stress‐related disorder symptoms such as fatigue, decreased performance, and impaired recovery from overtraining syndrome in sports medicine.^76^ The major symptoms and signs of overtraining were categorized^77^ as:

- physiological (chronic fatigue, decreased performance and muscular strength, muscle soreness, extended recovery time, increased oxygen uptake at physical loads, loss of appetite, and decreased body fat).
- psychological (difficulty concentrating, emotional instability characterized as restlessness and excitation followed by apathy and depression),
- immunological (immunosuppression characterized as decreased blood immunoglobulins and lymphocyte count, decreased chemotaxis of neutrophils, increased susceptibility to infection),
- biochemical (decreased free testosterone and raised cortisol levels, elevated lactate, and reduced hemoglobin levels in blood).

All of these symptoms of overtraining healthy subjects in stress as well as their overall health status are in line with a subpar health status^78^ known in TCM as “shanghuo” or “re‐qi” (upper fever, pathology fire, internal heat, or excessive energy associated with energy metabolism), which is characterized by a general decline in health, cut of energy, weakness, impaired physiological functions and adaptability (presumably Xie‐Huo in TCM), leading to the onset and progression of diseases.^79^

In other words, “shanghuo”^79^ is a state of decreased resistance (or increased susceptibility) leading to stress and progression of diseases. That is similar to low‐grade inflammation,^80^ resulting in and involving whole‐body systems such as the neuroendocrine‐immune (stress‐system), cardiovascular, and other systems.

According to TCM, the onset of disease is due to both external (wind, cold, heat, dampness, dryness, fire) and internal causes—excessive emotional activity induces the yin‐yang imbalance of the following seven emotions: joy, anger, anxiety, concentration, grief, fear, and fright. Bacteria, viruses, and chemicals are not considered to be causes. Most people whose health is not affected by external factors, but in whom excessive emotional activity causes a severe yin‐yang imbalance, experience blockage of qi and impairment of vital organ function. According to TCM theory, “shanghuo” caused by emotional stress can induce insomnia, depression, increase susceptibility to infectious diseases, and promote cardiovascular disease and tumor progression. Therefore, unsurprisingly the idea to prevent and treat stress‐induced disorders caused by a yin‐yang imbalance with prophylactic treatment using medicinal plants trace back to centuries (e.g, Weibing in China, Mibyeong in Korea,^81^ and Mibyou in Japan.^82^ Subsequently, the concepts underlying preventive treatment for subhealth by adaptogens (presumably “fu zheng” in TCM for strengthening body resistance or strengthening vital qi) were implemented in USSR under the names Medical Fitness, Farmacosanacia, and Valeology.^83^

According to TCM, the treatment of diseases must rectify harmony, and restore qi and the yin‐yang balance. It is the quality, quantity, and balance of qi that determine the state of health and lifespan. Food and air affect health; therefore, diet and breathing exercises are of primary importance. According to The Divine Husbandman's Classic of the Materia Medica, the earliest existing monograph of TCM prepared 4000 years ago, *P. ginseng* tonifies the primal qi and qi of all organs, particularly those of the lungs and spleen. Therefore, it has been indicated for deficiency of qi in patients with shallow breathing, shortness of breath, coldness of limbs, profuse sweating, or weakness and has been used to reduce the symptoms of stress and inflammation and delay aging.^84^

Medicinal plants are considered for the treatment of diseases and recovery of vital energy, which is believed to gradually dissipate throughout life. So, it is important to conserve it using diet, kung fu, breathing exercises, and herbal medicines. As an example, fatigue is due to qi deficiency, and *P. ginseng* (tonic herb) activates qi and therefore has nourishing effects in fatigue.^47^, ^85^, ^86^, ^87^, ^88^

In TCM, all known medicinal plants are divided into three categories: inferior, middle, and superior. The highest forms of medicine revered in China are the superior herbs (tonic herbs), which help everything to heal and nurtures life itself. Superior herbs are thought to possess restorative properties and are used as general tonics for the treatment of disease and in convalescence. The most well‐known broad action medicinal plant in TCM is ginseng.^89^, ^90^

The pharmacological activity of ginseng was first described in the 1st century by an unknown author. According to his records, ginseng improves mental activity and visual acuity, dispels pathogenic factors, enhances longevity with long‐term intake tonifying five vital organs of the body (spleen, lung, heart, kidney, and liver). According to other ancient regards written by Hongjing Tao (AD 456–536), ginseng can be used to enhance cognitive function; improve blood circulation; relieve thirst and feelings of solidity; and cure internal coldness, pain in the chest or abdomen, vomiting, and diarrhea. These and other beneficial effects of ginseng have also been described in other more complete and comprehensive medical textbooks including treatment for general weakness and fatigue.

“Kampo” (Traditional Japanese Academic Medicine) developed on the Japanese Islands from ca. 500 AD based on Ancient Chinese Medicine (ACM)—the common ancestor system of Japanese Kampo, Korean Medicine (KM), and Traditional Chinese Medicine (TCM). Subsequent independent developments and European influence in the 16th century resulted in a divergent cultural evolution establishing Kampo as an independent TMS distinct from other systems. Over the past centuries, fundamental philosophical differences have developed.^91^ Kampo is mostly based on the systematic collection of case histories—empirical knowledge of the effect of Kampo preparation. As Kampo is regulated by the Japanese government, Kampo prescriptions (as finished pharmaceutical products) are included in the Japanese Pharmacopoeia (JP) and covered by the national health insurance. Every Kampo formula is indicated for individuals with the same “symptom patterns” *(sho*), based on a pathological status of an individual.^91^

A special class of Kampo prescriptions with close similarity to the adaptogenic concept are the so‐called “support preparations” or *Hozai*. The term hozai is used to describe preparations that are applied to stop or partially reverse the symptoms of physical weakness and degenerative diseases. Hozai can be used in cases of typically geriatric ailments but also in any other case of physical decay.^92^, ^93^

The traditionally accepted explanation for the activity of Kampo medicines ‐ including Hozai —was summarized in the 18th century CE by the philosopher Yoshimasu Todo (1702–1773), who stated that curative and toxic effects are two phases of the same process; since diseases are triggered by uncontrolled poisoning, the patient has to be healed by a positive, challenging poisoning. This controlled poisoning initiates a regeneration reaction that removes toxicity from the body, thus restoring the patient's health.^94^ In this context, hozai and adaptogens are similar since adaptogens are eustressors (i.e., good stressors) acting as mild stress mimetics or stress‐vaccines that induce a stress‐protective response,^12^, ^14^, ^27^, ^60^, ^95^ which is in line with the basics of Kampo medicine.^91^ The relationship of the two concepts is illustrated by *P. ginseng* root—one of the classical USSR Adaptogens.^8^ This is an essential component drugs of most Hozai preparations (Table 4).^96^ The two major prescriptions of the hozai category are *Juzentaihoto* ^97^ and *Hochuekkito* ^98^ (Table 4).

**Table 4:** Crude drugs and their respective daily dosages (g) in the two traditional Kampo *Hozai* prescriptions^a^

*Juzentaihoto* (Japanese name) 十全大補湯	Hochuekkito (Japanese name) 補中益気湯
*Shi‐Quan‐Da‐Bu‐Tang* (Chinese name)	*Bu‐Zong‐Yi‐Qi‐Tang* (Chinese name)
*Sipjeondaebotang* (Korean name)	*Bojungikgitang* (Korean name)
Tonifying the Middle and Augment the Qi Decoction	Tonifying Decoction
https://kampo.ca/herbs-formulas/formulas/juzentaihoto/	https://kampo.ca/herbs-formulas/formulas/hochuekkito/
Ginseng Radix—3	Ginseng Radix—4
Astragali Radix—3	Astragali Radix—4
Glycyrrhizae Radix—1.5	Glycyrrhizae Radix—1.5
Angelicae sinensis Radix—3	Angelicae sinensis Radix—3
Atractylodis macrocephalae Rhizoma—3	Atractylodis macrocephalae Rhizoma—4
Paeoniae Radix—3	Bupleuri Radix—2
Cinnamomi Cortex—3	Jujubae Fructus—2
Ligusticum Rhizoma—3	Zingiberis Rhizoma—0.5
Sclerotium Poriae Cocos—3	Cimicifugae Rhizoma—1
Rehmanniae Radix preparata—3	Citri reticulatae Pericarpium—2

^a^ Corresponding daily dose is 7.5 g of dried extracts in representative finished pharmaceutical products (JP: The Japanese Pharmacopoeia). Both formulations are regarded as effective by the Japanese regulatory authorities and are available as finished pharmaceutical products of equal quality to traditional herbal medicinal products registered in the EU under coverage of the Japanese National Health Insurance.

Both formulations are mainly used in cases of geriatric ailments and physical decline.^93^ *Juzentaihoto* is also used for decubitus ulcers, radiation sickness, rheumatoid arthritis, supportive therapy in cancer, and to reduce adverse effects from surgical treatment and chemotherapy. The indications given by the Japanese national health insurance for *Hochuekkito* are related to general vigor, anorexia, myasthenia gravis, chronic gastritis, and atopic dermatitis.^99^, ^100^

The Western indications, for which hozai are most often used in Japan, are related to cachexia,^101^, ^102^ a loss of skeletal muscle mass that differs from weight loss due to malnutrition, anorexia nervosa, or anorexia due to depression or sarcopenia (aging‐related muscle loss).

*In conclusion*, shanghuo, a state of decreased resistance to stress can be treated with what—first in the Soviet/Russian literature—has been labeled adaptogenic plants. These will and increase the nonspecific resistance to stress; the yin‐yang balance, a synonym of homeostasis (see the next section of this chapter); and vital energy or qi, which has a similar meaning as adaptability or a state of nonspecific resistance. The concept of hozai is very similar to the adaptogenic concept, particularly in the context of their modes of action as eustressors (i.e., good stressors), and as mild stress mimetics or stress‐vaccines that induce a stress‐protective response; its systematic use in gerontology might be very beneficial, as has already been demonstrated in Japan.

The multipurpose use of adaptogens (ginseng) in numerous conditions suggests their nonspecific and normalizing effects in organisms. The traditional use of ginseng in billions of people for centuries is one important argument in favor of it being nontoxic, innocuous, and not influencing normal bodily functions more than necessary.

#### 2.2.2 Ayurveda

Ayurveda is a conventional medicinal system with varied treatments, which originated over 3 millennia ago in South Asia.^103^ In Ayurvedic philosophy, the central concept is the Tridosha theory suggesting that good health occurs when there is a dynamic balance between three fundamental dynamic forces or dosh as called Vata, Pitta, and Kapha.

o Vata is the combination of air and water, which is associated with the function of the nervous system. An imbalance leads to pain, sleeplessness, and inability to concentrate and stay on task.
o Pitta is the combination of fire and water, and is associated with bile, digestion, and metabolism.
o Kapha is the combination of water and earth, and is associated with mucous, lubrication, and transporting nutrients into the arterial system.

According to Ayurvedic theory, the life vital energy, Prana, comes from the air into the brain via respiration. Prana is settled in the brain and governs emotions, memory, and other functions of the mind. It also rules the functioning of the heart and enters the bloodstream to control all vital organs.

In Ayurveda, the plants known as rasayana are used as rejuvenating and for improving the overall health of anyone undergoing this treatment. The word rasayana literally means the path that rasa takes (rasa: the primordial tissue or plasma; ayana: path). According to Ayurveda, the qualities of rasa‐dhatu influence the health of other dhatus (tissues) of the body, as it is the most primary in function and works as the basic unit. Hence any medicinal plant or formulation that improves the quality of rasa (rasayanas), strengthen or promotes the health of all tissues of the body. Apart from promoting good health, increasing the ability to concentrate, improving memory and mood, an important effect of rasayana therapy is increasing resistance to diseases.^104^ The rasayana effect is not a specific pharmacological action, but rather a complex response operating through a comprehensive holistic mechanism of regulation of homeostasis.

Species most commonly used in Ayurveda as rejuvenating include:

o Ashwagandha—*Withania somnifera* (L.) Dunal
o Kalmegh—*Andrographis paniculata* (Burm. F.) Wall. Ex. Nees.
o Yasthimadhu (Licorice)—*Glycyrrhiza glabra* L.
o Satavari—*Asparagus racemosus* Willd
o Tulsi (Holy basil)—*Ocimum tenuiflorum* L. (syn.: *Ocimum sanctum* L.)
o Pipul (Pepper)—*Piper longum* L.
o Guduchi—*Tinospora cordifolia* Miers
o Amla—*Emblica officinalis* Gaertn
o Haritaki—*Terminalia chebula* Retz.

*W. somnifera* is used in Ayurveda toward promoting health and longevity, slowing the aging process, revitalizing the body, reducing anxiety, and creating a general sense of well‐being. These traditional applications of *W. somnifera* are due to a wide range of pharmacological effects observed in recent preclinical studies in animals and clinical trials in humans including anxiolytic, sedative, anti‐inflammatory, analgesic, immunomodulatory, antioxidant effects, cardiopulmonary, and hypotensive effects.^105^

*A. paniculata*, “the king of bitters,” is used in Ayurvedic and other traditional health care systems of India, China, and other Asian countries for numerous medicinal purposes, for example as an effective antipyretic treatment against a variety of infectious diseases including bronchitis, tonsillitis, tuberculosis, malarial and intermittent fever, urinary infection with difficult painful urination, dysentery, bacillary dysentery, colitis, dyspepsia, hepatitis, mouth ulcers, colic, otitis, vaginitis, pelvic inflammatory disease, chickenpox, carbuncles, sores, and eczema. The plant is effective for venomous snake bites, burns, and traumatic infection. Efficacy for prophylaxis and symptomatic treatment of upper respiratory infections such as the common cold, bronchitis uncomplicated sinusitis and pharyngotonsillitis, urinary tract infections, and acute diarrhea has been supported by clinical studies.^4^

The root of the liquorice plant (*Glycyrrhiza* sp.) is also oa well‐known rasayana drug in Ayurveda mainly due to anti‐inflammatory, antiviral, and antimicrobial activities.

In Ayurveda, *A. racemosus* is used as rasayana medicine and is acknowledged for promoting physical and mental health. Its wide range of therapeutic effects such as antitussive, antiplasmodial, anti‐leishmanial, antibacterial, hepatoprotective, diuretic, antiulcer, antidiarrheal, antenatal tonic, cardioprotective, anticancerous, antiepileptic, and antidepressant are likely associated with its immunomodulatory and adaptogenic activities.^106^, ^107^ However, many of these therapeutic claims go well beyond preventive medical concepts.

In Ayurveda, *P. longum* is used in hepatosplenomegaly, respiratory disorders including asthma, chronic cough, tuberculosis, skin disorders, piles, diabetes, and anemia. It is also beneficial in fever and infection including typhoid and has analgesic effects in dyspepsia, worm infestation, and abdominal pain. It is also reported to have aphrodisiac properties. *P. longum, P. nigrum*, and *Zingiber officinalis* are combined in the Ayurvedic formulation Trikatu, which is effective in several ailments. It increases the action of other drugs by increasing the bioavailability, as piperine is the main biomarker compound.^108^

In Ayurveda, Guduchi (*T. cordifolia*) is effective against various infections to boost immunity, especially in the convalescent period, as it has antipyretic, analgesic, and anti‐inflammatory properties. It is also useful for dyspepsia, anorexia, liver disorders, dysentery, and worms, and is prescribed for anemia, diabetes mellitus, gout, and rheumatoid arthritis.

In Ayurveda, *E. officinalis* is used for the treatment of peptic ulcer, dyspepsia, altered gastrointestinal motility (diarrhea, constipation, vomiting), and symptoms from pancreatitis, piles, liver disorders, diabetes, tuberculosis, and other lung infections. It has anti‐inflammatory and antistress effects. Regular intake of *E. officinalis* fruit has been advised for the general maintenance of health and preventive healthcare. External application is prescribed for alopecia or baldness, toothache, and ophthalmic conditions.^109^

*T. chebula* is considered as digestive and gives strength to tissues, particularly the sense organs. It purifies blood and has laxative and antipyretic actions. It is prescribed for dyspepsia, piles, hepatosplenomegaly, irritable bowel syndrome, and cardiac dysfunction. Triphala, a formulation containing equal parts of *E. officinalis, T. chebula*, and *T. bellerica*, is used as a laxative and general well‐being as it maintains the balance of Vata, Pitta, and Kapha.

Modern practices derived from Ayurveda are now classified as a type of complementary or alternative medicine, especially in the Global North.

*In conclusion*, the fundamental philosophy of Ayurvedic medicine, particularly in the context of homeostasis regulation of the stress‐system (neuroendocrine‐immune complex, see below), nonspecific resistance (vital life energy = prana), pharmacologically pleiotropic or polyvalent effects, and the antiaging effects of adaptogens is very similar to the concept of adaptogens.

#### 2.2.3 Impact of ancient Greece, Rome, and medieval TMS of middle Asia

Yunani or Unani is the term for Parsi‐Arabic traditional medicine as practiced in the Indian subcontinent, and in Muslim culture in central and southern Asia. The term is derived from Arabic Greek and has Hellenistic origin based on teachings of the Greek physicians Hippocrates, Dioscórides, and Galen Unani. It was further developed and enriched by Abu‐Ali Ibn Sina (Avicenna), Amirdovlat, and other medieval physicians and philosophers.^110^

For instance, Amirdovlat devoted considerable attention to those medicinal plants, which had antitoxic (lavender, marigold, ironwort) and tonic properties (birthwort, bryony). Amirdovlat used bryony, the sacred medicinal plant, as a panacea for all diseases to prevent premature aging and maintain good health and vitality.^111^, ^112^

In pre‐Christian times, the root of *Bryonia alba L*. was an occult object in Armenia (Loshtak in Armenian), where it was used as a drug for all diseases.^113^, ^114^ It has been referenced by the scientists of ancient Greece (Dioscórides, Hippocrates, Theophrastus), Rome (Celsius, Columella, Galen, Plinius), and Asia (Amirdovlat, Avicenna), and was studied in Jensen's 1914 thesis.^114^, ^115^ The *Bryonia* root has been used to treat a wide range of conditions and disorders including fatigue, gout, arthritis, rheumatism, neuralgia, pain. psoriasis, abscesses, allergies, leprosy, edema, bronchitis, pleurisy, asthma, tuberculosis, tonsillitis, lung inflammation, cough, influenza, fever, sciatica, ulcers, gastrointestinal diseases, liver diseases, cancer, hypertension, cardiovascular diseases, epilepsy, lockjaw, paralysis, hysteria, madness, sleeplessness, and impotence. It has also been used as a laxative, cathartic, lactogenic, anthelmintic, diuretic, expectorant, and to induce abortion, as well as a cosmetic to remove spots, pimples, warts, blackheads and bruises; to prevent allergic reactions and for the prevention of hair loss.^110^, ^111^, ^114^

*Bryonia* extract was integrated in official medicine as a tonic and adaptogenic drug in Armenia, Russia, Ukraine, and Belorussia in the 1990s of the XX century and the first decade of the XXI century.^114^ Preparations from *Bryonia alba* L. root extract (“Loshtak” tablets) were registered as medicines by the Russian Federation in 2002, Belarus in 2003, Ukraine in 2007, and Armenia in 1992 and 2003 as an adaptogen and tonic in asthenia; agent for decreased resistance to infections; maintenance of working capacity, coordination, and mental activity; and prevention of stress, radiation‐ and chemotherapy‐induced toxicity and disorders, and so forth.

*In conclusion*, experiences in ancient Greece, Rome, and Medieval TMS of Middle Asia, particularly regarding the multitasking effects of medicinal plants as a panacea for all diseases can be expressed using the modern concept of adaptogens, and their benefits at low doses to prevent premature aging and maintain good health and vitality.

#### 2.2.4 European traditions and core rational elements of homeopathy

The basic idea of homeopathy assumes that a substance at a high dose causes the symptom of disease in healthy subjects, while curing similar symptoms in illness if applied at a low dose.

Homeopathic preparations are made from ingredients which, in undiluted form, cause symptoms similar to the disease they aim to treat. These ingredients are repeatedly diluted, with shaking at each stage (Table 5). Homeopaths consider that this technique prevents side effects, enhances the ability of preparations to amplify a response, and generates curative properties, even for ingredients that are chemically inactive or so significantly diluted that none of the original material remains. While high‐potency preparations (i.e., highly diluted ones) clearly cannot be evaluated using bioscientific concepts and methods, lower potency ones may well exert relevant pharmacological and toxicological effects.

**Table 5:** Dilution scales and homeopathic potency

Dilution scales	
Mother tincture	Homeopathic potency
1 vol of tincture + 9 vol solvent = D1	1X
1 vol of D1 + 9 vol solvent = D2	2X
1 vol of D2 + 9 vol solvent = D3	3X
1 vol of tincture + 99 vol solvent = C1	1H
1 vol of C1 + 99 vol solvent = C1	2H

*Note*: Preparations obtained by dilution of 1 M solution (6.02 x 1023 molecules per L) in potencies higher of D24 do not actually contain a single active molecule.

Homeopathic preparations are generally not tested and regulated under the same laws as conventional drugs. Usage varies from only 2% of people in Britain and the United States using homeopathy in any 1 year, to 15% in India, where homeopathy is now considered part of its traditional medicine. Homeopathic medicines are generally considered safe, with rare exceptions.

However, homeopaths have been criticized for putting patients at risk by advising them to avoid conventional medical treatments.

According to homeopathic theory, the efficacy and safety of the same plant significantly depends on when and where it was collected, and how it was processed. For example, freshly collected summer roots of *Bryonia* are used in the homeopathic tincture *Acofit* and is indicated in lumbago, neuromyelitis, and radiculomyositis, whereas 20% of ethanolic extract and dried powder of the roots are recognized as a treatment for bronchitis, pleurisy, asthma, whooping cough, and other inflammatory disorders.^114^, ^115^, ^116^, ^117^, ^118^ Homeopathic tablets and pellets are used in the United States, England, France, Germany, and Russia for the treatment of rheumatic pain and headache; acute inflammation of the pleura and abdomen; and fever and viral infections (mainly in combination with *Aconitum, i.e. Bryaconel Heel*, 1994). Various preparations of *Bryonia* roots are used to relieve muscle pain and diminish the symptoms of asthma and epilepsy.^116^, ^119^

In addition to homeopathy, other traditions also pay close attention to self‐healing and coping with adverse situations. Anthroposophical medicine is a complementary medical tradition founded in the 1920s by Rudolf Steiner,^120^ who advocated for the use of *Viscum album* L. (the European white‐berry mistletoe) in cancer.^121^ It is a holistic approach to medicine focusing on ensuring that the conditions for health are present in a person.

Anthroposophical therapies are intended to enhance an organism's ability to heal in line with the adaptability concept and the concept of adaptive homeostasis, as explained below.

*V. album* L., an obligate hemiparasite plant growing on apple, pear, plum, hawthorn, beech, willow, poplar, maple, sweetgum, oak, almond, elm, pine, spruce, juniper, and eucalyptus, exhibits immunostimulatory, anti‐inflammatory, analgesic, antioxidant, antiglycemic, antihypertensive, and neuroprotective properties.^122^ In allopathic doses, mistletoe preparations (fresh juice, tinctures, and decoctions of various parts) are used in various countries (Armenia, Russia, Ukraine, Bulgaria, the Czech Republic) to treat cough, broken bones, diarrhea, rheumatism, gout, inflammation of lymphatic glands, wounds, and ulcers, as well as hypotensive, antiatherosclerotic, antiosteoarthritis, analgesic, sedative, and antiepileptic remedies.^123^ It is worth noting that mistletoe growing on different trees are used for different purposes. Thus, mistletoe growing on the willow is mainly used as a sedative, whereas mistletoe growing on the pear is used in cardiovascular medicine, and the one growing on the hawthorn is used as a hypotensive drug.^123^

In homeostatic doses, the mistletoe preparations Iscador, Eurixor, Helixor, Abnoba‐viscum, and Isorel standardized for the content of mistletoe lectin 1 (1 ng/kg) are widely used in Europe as alternative adjuvant therapies against colon, oral, lung, pancreatic, and breast cancers.^124^ The mistletoe extracts boost immunity, delay tumor progression, improve the quality of life, and increase survival and lifespan of cancer patients by helping with coping, fatigue, sleep, exhaustion, energy, nausea, vomiting, appetite, depression, anxiety, the ability to work, and emotional and functional well‐being.^125^, ^126^, ^127^, ^128^ Mistletoe treatment also alleviates the adverse effects from chemotherapies.^129^

*In conclusion*, the same substance can have dose‐dependent reversal effects.^130^ In small doses, it can activate defence systems and exhibit beneficial/curative effects, while in high doses, it can inhibit the defence system and be harmful for the organism. The “bell shape” dose‐effect relationship is common for adaptogens, which have high therapeutic indices (effective dose: toxic dose ratio). In addition, toxic medicinal plants in small doses activate the body's defence systems, particularly the immune system, to cope with cancer and other diseases associated with suppressed immunity. Adaptogens similarly activate the body's defence systems, but at doses not toxic for humans.

### 2.3 Physiological background on the adaptogenic concept

The concept of adaptogens is based on Hans Selye's theory of stress and homeostasis. The word “stress” is commonly used in numerous conditions and has quite different meanings in daily life. In this review, we used commonly accepted definitions of stress, homeostasis, adaptive stress response, and adaptive homeostasis^131^ (Table 6). Repeated mild exposure or low doses of stress induce the increased resistance of cells and organisms to subsequent stress exposure, resulting in an adaptation favouring survival. This phenomenon of adaptation to repetitive low‐level stress was first described by Hans Selye in 1936.

**Table 6:** Definitions of stress, stress system, homeostasis, adaptation, adaptedness, adaptability, resilience, adaptive homeostasis, adaptive stress response (hormesis), adaptive stress system, and adaptive signaling pathways

**Figure 1:**
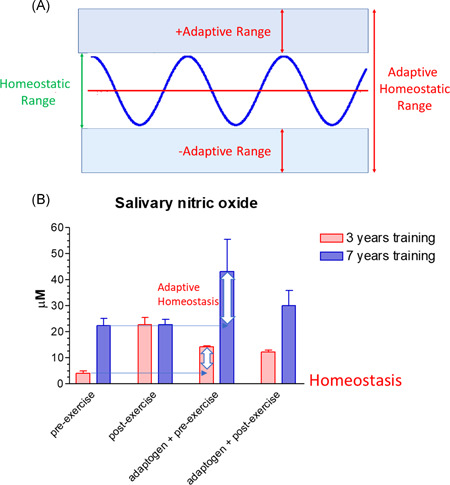
(A) Adaptive homeostasis was defined as the transient reversible adjustments of the homeostatic range in response to exposure to signaling molecules or events. Any biological function or measurement oscillate around a mean or median, within a homeostatic range that is considered a “normal” or physiological, upgraded from Reference [131]. (B) Adaptogens and physical exercise adjust the homeostatic range of salivary nitric oxide. Effects of physical exercise and androgens on the nitric oxide level in saliva of athletes regularly trained for 3 and 7 years^141^ [Color figure can be viewed at wileyonlinelibrary.com]

Stress is a state of threatened homeostasis,^132^ depending on severity and duration, stress can have quite a different impact on the organism—from beneficial to harmful: chronic eustress (too little stress), acute stress (optimum stress) initiate beneficial adaptive stress response, while when stress increases beyond a certain level ‐ acute distress (too much stress), and chronic stress (burnout)—it leads to harmful health effects and can cause numerous diseases. In this context, adaptogens act like chronic eustress activating adaptive stress response, resilience and overall survival.
Stress system is the neuroendocrine–immune complex, Adaptive stress system includes all physiological systems involved in the process of adaptation to stress.^133^
Homeostasis is a complex dynamic equilibrium/steady state, maintained by coordinated physiological processes in the organism.^132^, ^134^ In other words, homeostasis is the ability of a living organism or cell to maintain the state of internal balance despite changes in the conditions around it, while stress—is temporary inability to maintain this steady state.
Adaptation as an active process of responding to challenges which includes behavioral, physiological, structural, and genetic changes upon environmental impacts that are beyond the biologically adequate ranges.^135^
Adaptedness is a result of adaptation process when a positive outcome, that is, survival and reproduction, is achieved in the face of adversity. Adaptedness is a state that has a capacity for adaptation.^136^
Adaptability is an ability of an organism to alter itself or its responses to the changed circumstances or environment. Adaptability shows the ability to learn from experience and improves the fitness of the learner as a competitor.^137^
Resilience is the ability to maintain or quickly return to a stable physical and psychological equilibrium despite experiencing stressful events.^138^
Adaptive homeostasis is defined as the transient reversible adjustments of the homeostatic range in response to exposure to signaling molecules or events.^131^ Adaptive homeostasis is the cellular or organismal capability to adjust the homeostatic range in response to herbal adaptogens.^17^ In this context, adaptogens increase homeostatic range to the level of adaptive homeostasis activating adaptive stress response resulted in increased resilience and overall survival, Figure 1.
General adaptation syndrome–three phase response including nonspecific reactions (thymus atrophy, adrenal, hyperplasia, stomach ulceration, increased secretion of cortisol and catecholamines, etc.) of organisms evoked to stress: (i) alarm phase, (ii) phase of nonspecific resistance, following which symptoms disappear, and (iii) phase of exhaustion, when the same symptoms reappear, followed by death.^139^, ^140^ Adaptive stress response (hormesis) is the ability of a cell, tissue, or organism to better resist stress damage by prior exposure to a lesser amount of stress is known as adaptive response. It is observed in all organisms in response to a number of different cytotoxic agents.

Survival of organisms and resistance to stress depends on adaptability, and adaptive homeostasis is the threshold that determines an organism's innate tolerance to a given level of stress (Figure 1).

In recent years our understanding of mechanisms underlying the health benefits of natural dietary compounds has improved considerably. Based on modern concepts, plants synthesize in their most susceptible parts (flowers, roots, and leaves) special secondary metabolites for self‐protection against microorganisms, insects, and other pests, as well as to mitigate harmful environmental conditions.^142^, ^143^, ^144^ In animals that use plants as their primary nutrition multiple mechanisms to counteract the potentially poisonous effects of phytotoxins have evolved. These natural compounds are not noxious in humans at lower doses but are able to induce mild cellular stress responses.^145^ The ability of plant secondary metabolites to activate the adaptive cellular stress response pathway in human body is one of their essential mechanisms of action.^142^, ^144^

**Figure 2:**
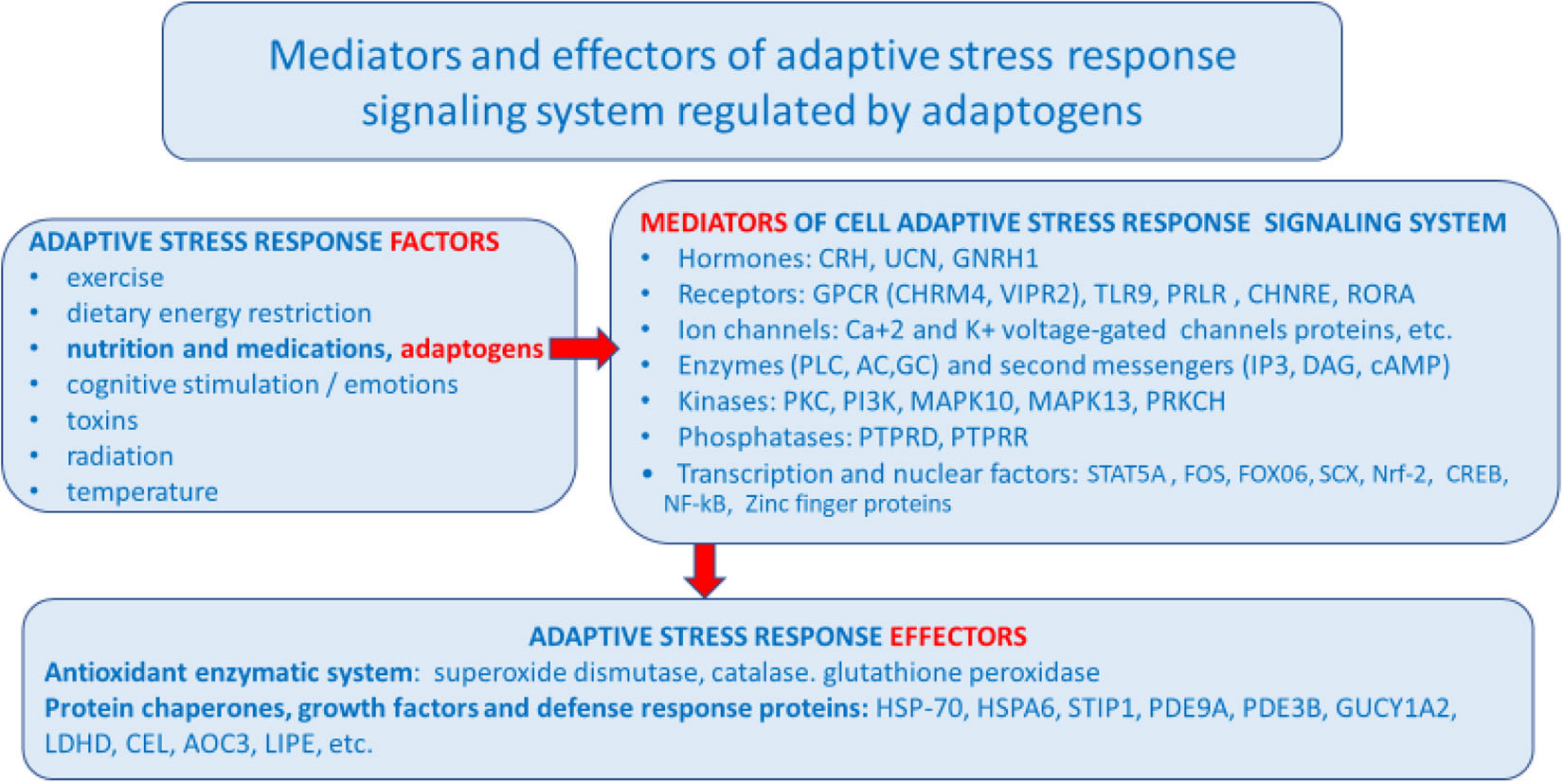
Adaptive stress response factors, mediators, and effectors (updated and adapted from Reference [143] and authors’ drawings.^17^ Adaptive stress response involves activation of intracellular and extracellular signaling pathways and increased expression of antiapoptotic proteins, neuropeptides, antioxidant enzymes, and defense response of an organism resulting in increased survival. One basic mechanism of action of adaptogens, that is, that they activate adaptive cellular stress response pathways in humans’ brain cells [Color figure can be viewed at wileyonlinelibrary.com]

This phenomenon has been categorized as hormesis or as adaptive stress response, pre‐conditioning.^146^, ^147^ The multiple mediators of the stress signaling system (the neuroendocrine–immune complex) including different growth factors, antioxidants, and stress‐resistant proteins such as heat shock proteins (Hsps) are involved in stress‐induced responses of the innate and adaptive defence systems.^17^, ^148^, ^149^ We suggest that adaptogens are the first line of plant secondary metabolites activating adaptive stress response pathways^17^ (Figure 2).

Adaptive stress response is important in cell maturation, with initiation by mild stress of mechanisms of repair and maintenance to protect cells against subsequent stresses, while chronic stress induce progressive failure of these mechanisms, leading to cellular senescence, aging, and death.^150^ With cellular maintenance on overdrive, the organism can continue to protect himself from chronic inflammation, which causes a range of serious illnesses, particularly aging‐related diseases.

**Figure 3:**
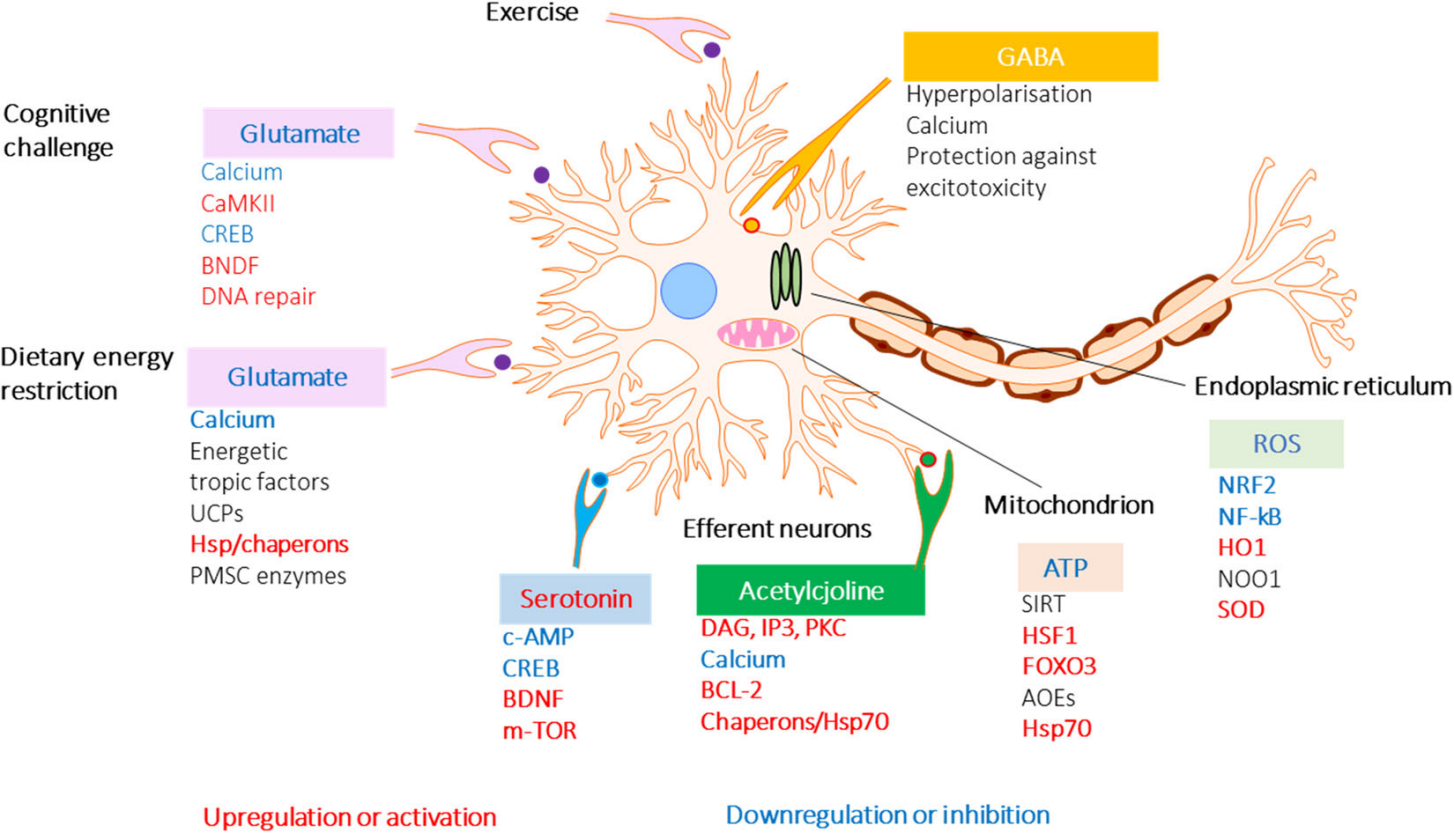
Effects of adaptogens on adaptive stress response signaling pathways that promote synaptic plasticity and protect neurons against degeneration. Illustration of a glutamatergic neuron receiving excitatory signals from neurons activated in response to intellectual tasks, exercise, and dietary energy restriction. Postsynaptic receptors for glutamate, acetylcholine, and serotonin, are activated to trigger intracellular signaling pathways and transcription factors that activate the expression of neuroprotective proteins including antiapoptotic proteins, brain‐mitochondrial uncoupling proteins (UCPs), and derived neurotrophic factor (BDNF). BDNF activates neuronal growth by stimulating the mammalian target of rapamycin (mTOR). Mild cellular stress resulting from dietary energy restriction and oxidative stress (ROS) activates adaptive stress response pathways including those that upregulate antioxidant enzymes (AOEs) and protein chaperones. CREB, cyclic AMP response element‐binding protein; CaMKII, calcium/calmodulin kinase II; DAG, diacylglycerol; FOXO3, forkhead box protein O3; HO1, heme oxygenase 1; HSF1, heat shock factor 1; IP3 PKC, inositol trisphosphate 3 protein kinase C; NF‐B, nuclear factor B; NRF2, nuclear regulatory factor 2 NQO1, NAD(P)H‐quinone oxidoreductase 1 (updated and adapted from Reference [59] and from authors’ drawings^16^ [Color figure can be viewed at wileyonlinelibrary.com]

**Figure 4:**
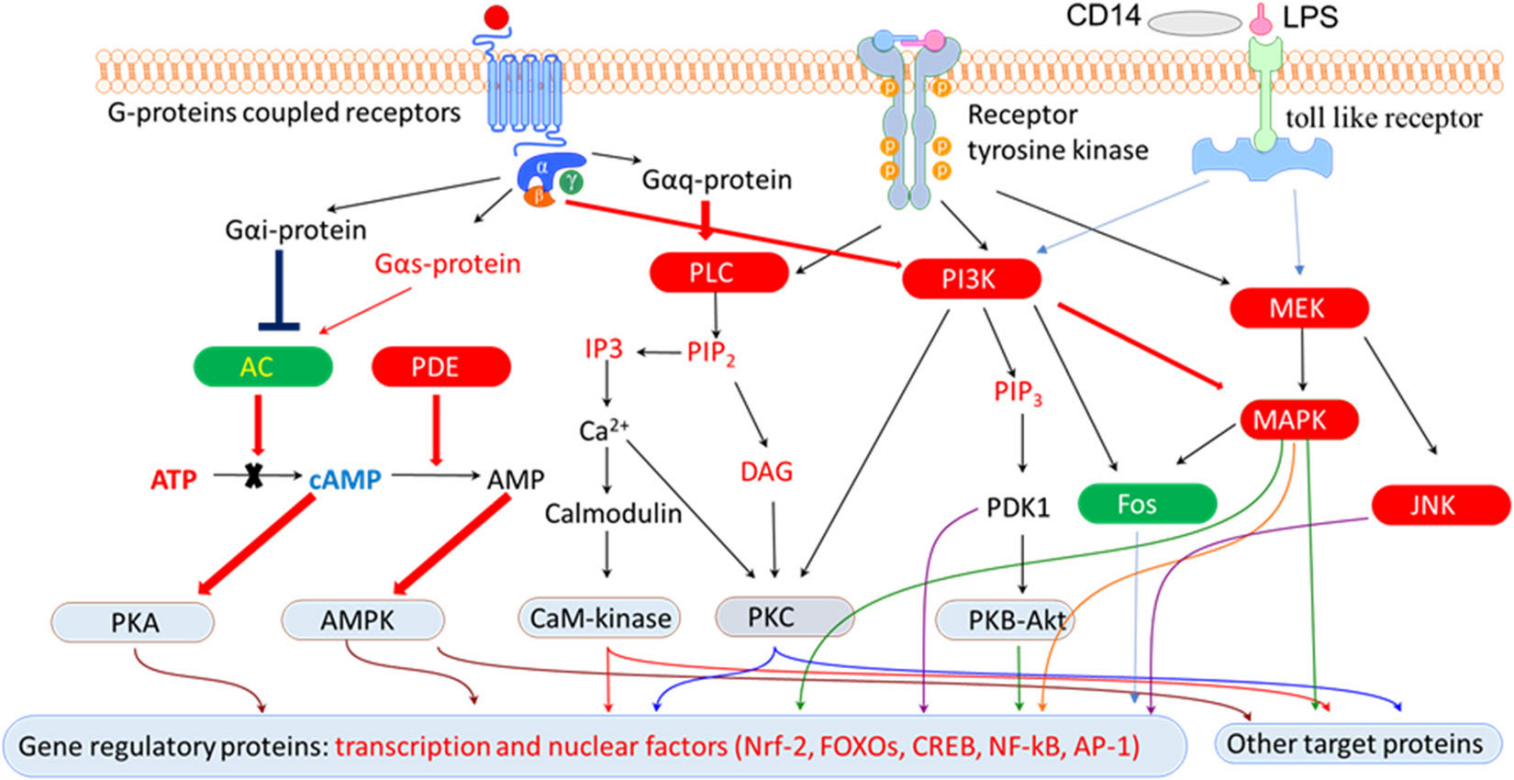
Effects of adaptogens on adaptive stress response intracellular signaling pathways (updated from authors’ drawings^17^). Activation of the PI3K/AKT/mTOR signaling pathway positively regulates cell cycle, proliferation, neural long‐term potentiation (memory cognitive functions and longevity. AC, adenylate cyclase; AMPK, 5' AMP‐activated protein kinase; AP‐1, activator protein 1 transcription factor; CREB, cyclic AMP response element‐binding protein; DAG, diacylglycerol; Fos, Fos proto‐oncogene, AP‐1 transcription factor subunit; FOXOs, forkhead box proteins; IP3, inositol 1,4,5‐trisphosphate; JNK, c‐Jun N‐terminal kinases; MaM‐kinase, Ca^2+^/calmodulin‐dependent protein kinase II; MAPK–MEK (MAPK/ERK), mitogen‐activated protein kinases; NF‐κB, nuclear factor kappa‐light‐chain‐enhancer of activated B cells; NRF2, nuclear regulatory factor 2; PDE, 3',5'‐cyclic‐AMP phosphodiesterase; PI3K, phosphoinositide 3‐kinase; PIP3, phosphatidylinositol (3,4,5)‐trisphosphate; PIP2, phosphatidylinositol (4,5)‐bisphosphate; PKA, cAMP‐dependent protein kinase; PKB‐Akt, serine/threonine‐specific protein kinase; PKC, protein kinase C; PLC, phospholipase C [Color figure can be viewed at wileyonlinelibrary.com]

The adaptive stress response is a survival mechanism. All functions of the body systems (e.g., cardiovascular, immune, nervous, endocrine, gastrointestinal digestive) are regulated by about 30,000 genes and fragments of DNA, which are located in the nucleus of every single cell. The activity of genes depends on the signals/stimuli received from numerous receptors and various proteins located on the outside surface of the cell membrane. The receptors specifically trigger signals from extracellular molecules—stressors (Figure 3) and transfer the signals to genes via many signaling cascades (adaptive signaling pathways), which can interact and influence each other in a complex molecular network (Figure 4). Collectively, this stimulus‐response system is known as the adaptive stress response system of the body responding to environmental stress.^16^, ^58^, ^143^, ^148^, ^149^, ^151^

*In conclusion*, the adaptive stress response is a survival mechanism that includes the genetic response to environmental mild stressors. The mild stressors include exercise, calorie restriction, and adaptogens, which activate adaptive signaling pathways of the adaptive stress system to boost the body's cellular maintenance functions into high gear with cells having a more efficient response. Adaptogens trigger the adaptive stress response to reduce chronic inflammation (inflammaging) and promote healthy aging.

## 3 ADAPTOGENIC PLANTS AND THEIR ACTIVE COMPOUNDS

The principal active constituents of adaptogenic plants (as investigated to date, Table 7) can be divided into three main chemical groups^16^:

o compounds with a tetracyclic skeleton like cortisol and testosterone—terpenoids ginsenosides, sitoindosides, cucurbitacines, and withanolides,
o structural analogues of catecholamines or tyrosine—lignans (schizandrin B from *S. chinensis*, eleutheroside E from *E. senticosus*), phenylpropane derivatives (rosavin from *R. rosea* and syringin from *E. senticosus*), phenylethane derivatives (tyrosol and salidroside from *R. rosea*),
o structural analogues of resolvins^152^—oxylipins (polyhydroxylated polyunsaturated fatty acids^16^).

**Table 7:** List of the plants reported to have antistress (adaptogenic) activity and used in traditional medicinal systems as rejuvenating medicinal plants, *qi* tonics, *rasayanas*, or restoratives

	Plants reported in Literature as adaptogens (1)* and for antistressa		References supporting such use in specific medical systems	
		Reference^b^	TCM—*qi* tonifying	Ayurveda
1	*Aegle marmelos* (L.) Corrêa	[153]		[154]
2	*Ajuga turkestanica* (Regel) Briq.	[155]		
3	*Albizia julibrissin* Durazz.	[156]		
4	*Alstonia scholaris* (L.) R. Br.	[157]		[158]
5	*Allium sativum* L.	[159]		
6	*Andrographis paniculata* (Burm.f.) Nees	[160, 161]		[162]
7	*Annona muricata* L.	[163]		
8	*Aralia elata* (Miq) Seem.	[164]		
9	*Aralia elata var. mandshurica* (Rupr. & Maxim.) J.Wen *(*syn. *Aralia mandshurica* Rupr. & Maxim)	[165, 166, 167]		
10	*Aralia cordata var. sachalinensis* (Regel) Nakai *(*syn.*Aralia schmidtii*Pojark.)	[165]		
11	*Argyreia nervosa* (Burm. f.) Bojer (syn. *Argyreia speciosa* (L. f.) Sweet)	[168]		[168]
12	*Asparagus racemosus* Willd.	[169]		[170, 171]
13	*Azadirachta indica* A. Juss.	[172]		[173]
14	*Bacopa monnieri* (L.) Wettst.	[174]		[175, 176]
15	*Bergenia crassifolia* (L.) Fritsch	[177, 178, 179]		
16	*Boerhaavia diffusa* Brandegee	[180]		[181]
17	*Bryonia alba* L.	[12, 114]		
18	*Butea monosperma* (Lam.) Taub.	[182]		[183]
19	*Caesalpinia bonduc* (L.) Roxb.	[184]		[185]
20	*Cannabis sativa* L.	[186]		[187]
21	*Carum carvi* L.	[188]		[188]
22	*Centella asiatica* (L.) Urb.	[189]		[190]
23	*Chlorophytum borivilianum* Santapau & R.R.Fern.	[191, 192]		[193, 194]
24	*Chrysactinia mexicana* A. Gray	[195]		
25	*Cicer arietinum* L.	[196]		[170]
26	*Clematis alpina subsp. sibirica* (L.) Kuntze (syn. *Atragene sibirica* L.)	[197]		
27	*Cnestis ferruginea* Vahl ex DC.	[198]		
28	*Codonopsis pilosula* (Franch.) Nannf.	[10]	[199, 200]	
29	*Convolvulus pluricaulis* Chois	[201]		[190]
30	*Curculigo orchioides* Gaertn.	[202]	[203, 204]	[204]
31	Curcumin from Turmeric *(Curcuma longa*)	[205]		[204]
32	*Dioscorea deltoidea* Wall. ex Griseb.	[206]		
33	*Diospyros malabarica* (Desr.) Kostel. (Syn. *Diospyros peregrina* (Gaertn.) Gürke)	[207]		
34	*Elaeagnus rhamnoides* (L.) A.Nelson. (Syn.*Hippophae rhamnoides* L.)	[208, 209]	[210]	
35	*Eleutherococcus senticosus* (Rupr. & Maxim.) Maxim.	[8, 165, 166]		
36	*Eleutherococcus sessiliflorus* (Rupr. & Maxim.) S.Y. Hu (syn *Acanthopanax sessiliflorus* (Rupr. & Maxim.) Seem.)	[8, 166]	[211, 212]	
37	*Emblica officinalis* Gaetrn.	[170]		[213, 214]
38	*Eucommia ulmoides* Oliv.	[215]	[216, 217]	
39	*Evolvulus alsinoides* (L.) L.	[218, 219, 220]		[218, 219]
40	*Fagopyrum esculentum* Moench	[221]		
41	*Firmiana simplex* (L.) W. Wight (Syn *Sterculia plantanifolia* L.)	[222]		
42	*Gentiana pedicellata* (D.Don) Wall	[223]		
43	*Ginkgo biloba* L.	[224]		
44	*Glycyrrhiza glabra* L.	[193, 225]	[226]	[193, 227]
45	*Hebanthe eriantha* (Poir.) Pedersen (Syn.*Pfaffia paniculata* (Mart.) Kuntze)	[228]		
46	*Heteropterys aphrodisiaca Machado*	[229]		
47	*Heteropterys tomentosa A.Juss*.	[230]		
48	*Hibiscus cannabinus *L.	[231]		
49	*Holoptelea integrifolia* Planch	[232]		[233]
50	*Hoppea dichotoma* Willd.	[234]		
51	*Hypericum perforatum* L.	[235]		
52	*Justicia diffusa* Willd. (Syn *Rostellularia diffusa* (Willd.) Nees.)	[236]		
53	*Lagenaria siceraria* (Molina) Standl.	[237]		
54	*Lepidium meyenii* Walp. (Syn. *Lepidium peruvianum* G.Chacón)	[238]		
55	*Marantodes pumilum* (Blume) Kuntze. (Syn.*Labisia pumila* (Blume) Mez)	[239]		
56	*Melilotus officinalis* (L.) Pall.	[240]		
57	*Mitragyna inermis* (Willd.) Kuntze (Syn *Mitragyna africana* (Willd.) Korth.)	[241]		
58	*Momordica charantia* L.	[242]		
59	*Morus alba* L.	[243]		
60	*Mucuna pruriens* (L.) DC.	[244]		[190]
61	*Murraya koenigii* (L.) Spreng.	[245]		
62	*Mussaenda frondosa* L.	[246]		
63	*Nelumbo nucifera* Gaertn.	[247]		[248]
64	*Nigella sativa* L.	[249]		
65	*Ocimum tenuiflorum* L. (Syn.*Ocimum sanctum* L.)	[250, 251, 252]		
66	*Oplopanax elatus* (Nakai) Nakai (Syn. *Echinopanax elatum* Nakai)	[165, 166, 253]		[165]
67	*Panax ginseng* C.A.Mey.	[8, 165, 187, 224]	[84, 85, 254]	
68	*Panax notoginseng* (Burk.) FH Chen			[107]
69	*Panax pseudoginseng* Wall.	[255]		[256]
70	*Pandanus odorifer* (Forssk.) Kuntze (Syn.*Pandanus odoratissimus* L.f.)	[257]		
71	*Paullinia cupana* Kunth	[258]		
72	*Putranjiva roxburghii* Wall. (Syn. *Drypetes roxburghii* (Wall.) Hurus.)	[259]		
73	*Piper longum* L.	[260, 261]		[170, 261]
74	*Polyalthia cerasoides* (Roxb.) Bedd.	[163, 262]		
75	*Polyscias filicifolia* (*C.Moore ex E.Fourn*.) *L.H.Bailey*	[263]		
76	*Potentilla alba* L.	[264]		
77	*Prunella vulgaris* L.	[265]		
78	*Psidium guajava* L.	[266]		
79	*Ptychopetalum olacoides* Benth.	[267]		
80	*Pueraria tuberosa* (Roxb. ex Willd.) DC.	[268]		
81	*Rhaponticum carthamoides* (Willd.)Iljin (Syn. *Leuzea carthamoides* (Willd.) DC.)	[8, 269]		
82	*Rhodiola crenulata* (Hook.f. & Thomson) H.Ohba	[270, 271]		
83	*Rhodiola heterodonta* (Hook. f. & Thomson) Boriss.	[272, 273]		
84	*Rhodiola imbricata* Edgew.	[271, 274]		
85	*Rhodiola rosea* L. [today classed as *Sedum roseum* (L.) Scop.]	[8, 21, 66, 72, 275, 276]	[277]	[278]
86	*Rubia cordifolia* L.	[279]	.	[280]
87	*Salvia miltiorrhiza* Bunge	[281]	[282]	
88	*Schisandra chinensis* (Turcz.) Baill.	[18, 64, 69, 165, 283]	[284, 285]	
89	*Scutellaria baicalensis* Georgi	[286]	[287]	
90	*Serratula tinctoria* L. (Syn.*Serratula inermis Poir*.)	[288]		
91	*Sida cordifolia* L.	[289]		[289]
92	*Silene italica* (*L*.) *Pers*.	[290]		
93	*Sinomenium acutum (Thunb*.) *Rehder & E.H.Wilson*	[291]		
94	*Solanum torvum* SW.	[292]		
95	*Serratula coronate* L.	[293]		
96	*Sutherlandia frutescens* (L.) R.Br.	[294]		
97	*Syzygium aromaticum* (L.) Merr. & L.M.Perry. (Syn. *Eugenia caryophyllus* (Spreng.) Bullock & S.G.Harrison)	[295]		[185]
98	*Terminalia chebula* Retz.	[296]	[297]	[170, 193]
99	*Tinospora sinensis* (Lour.) Merr. (Syn.*Tinospora cordifolia* (Willd.) Miers, Syn *Tinospora malabarica* (Lam.) Hook. f. & Thomson)	[298, 299]		[170, 300]
100	*Tribulus terrestris* L.	[301]		[301]
101	*Trichilia catigua* A.Juss.	[229]		
102	*Trichopodium zeylanicum* (Gaertn.) Thwaites (Syn.*Trichopus zeylanicus* Gaertn.)	[302, 303]		[302, 303]
103	*Trigonella foenum‐graecum* L.	[304, 305, 306]		[307]
104	*Tylophora indica* (Burm. f.) Merr.	[308]		
105	*Turnera diffusa* Willd. ex Schult.	[229]		
106	*Uncaria tomentosa* (Willd. ex Schult.) DC.	[309]		
107	*Vitis vinifera* L.	[310]		[310]
108	*Withania somnifera* (L.) Dunal	[232, 311, 312, 313]		[170, 314, 315, 316, 317, 318]
109	*Zingiber officinale* Roscoe	[319]		

b Updated from References [16, 183].

The number of plants reported as being adaptogenic has increased exponentially during the past decades. However, it should be emphasized that only a few comply with the most important criterium—exhibiting multitarget effects on the neuroendocrine‐immune system. These effects include triggering intracellular and extracellular adaptive signaling pathways that promote cell survival and organismal resilience in stress; and regulating metabolism and homeostasis via effects on the expression of stress hormones (corticotropin‐ and gonadotropin‐releasing hormones, urocortin, cortisol, melatonin, Hsp70, and neuropeptide Y) and their receptors.^16^, ^17^, ^18^, ^19^, ^20^, ^28^

Various adaptogens and their active principles—for example, salidroside,^320^, ^321^, ^322^, ^323^, ^324^, ^325^, ^326^ schisandrin A,^327^ schisandrin B,^328^ withaferin A,^329^, ^330^, ^331^, ^332^, ^333^, ^334^ Ginsenoside 20(S)‑Rg3,^335^ Ginsenoside 20(S)‐Rh2,^336^ compound K,^337^, ^338^ and 20(S)‐25‐methoxy‐protopanaxatriol^339^, ^340^—exhibit anticancer effects in various in vitro and in vivo models of breast, colorectal, prostate, hepatic, and intestinal cancers, and so forth by interacting with multiple intracellular signaling pathways, including the inhibition of proinflammatory pathways, such as the ERK/MAPK^341^ and STAT3 signaling pathways.^320^, ^321^, ^322^, ^323^, ^324^, ^325^, ^326^, ^327^, ^328^, ^329^, ^330^, ^331^, ^332^, ^333^, ^334^, ^335^, ^336^, ^337^, ^338^, ^339^, ^340^

It was found that compound K, an intestinal microbiome metabolite of ginsenoside Rb1,^342^ one of the major ginsenosides of Panax ginseng, has much stronger cancer chemopreventive activity than its precursor (Rb1 in HCT‐116 and HT‐19 human colorectal cancer cell lines), suggesting that Rb1 may have potential clinical significance in the prevention of inflammatory‐associated colorectal cancer^343^ because of the regulation of the microbiome balance and compound K.^343^, ^344^

*R. rosea* extracts and the active compound salidroside decrease the growth of bladder cancer cell lines via the inhibition of the mTOR pathway and induction of autophagy.^345^ Salidroside was shown to exhibit antioxidant, anti‐inflammatory, and anticancer effects in human breast cancer in vitro and in vivo experimental models.^346^ Salidroside treatment significantly inhibits MCF‐7 breast cancer cell proliferation, colony formation, migration, invasion, apoptosis, and cell‐cycle arrest at the G0/G1 phase in vitro and significantly suppressed tumor growth in vivo.^346^

In vitro and in vivo experiments demonstrated that salidroside enhances the chemotherapeutic effect of apatinib in gastric cancer.^347^ Ginseng potentiates the effects of chemotherapeutic agents via synergistic activities, supported by cell‐cycle evaluations, apoptotic observations, and computer‐based docking analysis.^348^

Finally, the results of many studies suggest that adaptogens might be useful for the prevention of liver cancer because of the upregulation of Nrf2 signaling, followed by the induction of the antioxidant and phase II detoxifying engines, for example, induction of the phase II detoxification enzyme NQO1 in hepatocarcinoma cells by lignans of *S. chinensis*, tigloylgomisin H (TGH), and angeloylgomisin H (AGH), which have exhibited a relatively high chemoprevention index (10.80 and 4.59, respectively).^349^

## 4 CURRENT AND PROSPECTIVE USE OF ADAPTOGENS IN STRESS‐INDUCED AND AGING‐RELATED DISEASES

Stress‐protective and stimulating effects are characteristic and common pharmacological effects of adaptogens,^73^, ^350^, ^351^ which have been observed in many animals and humans’ studies. The effects of adaptogens on cognitive functions and physical endurance in stress are summarized in several reviews.^10^, ^22^, ^26^, ^27^, ^350^, ^352^

The main difference between adaptogens and conventional stimulants such as caffeine and amphetamine is that after prolonged use, the latter can cause the user to develop both tolerance and addiction (Table 8).^27^, ^352^

**Table 8:** The differences in properties between adaptogens and other stimulants

	Stimulants	Adaptogens
Stress protective (neuro‐, hepato‐, cardio‐protective)	No	High
Recovery process after exhaustive physical load	Low	High
Energy depletion	Yes	No
Performance in stress	–	Increased
Survival in stress	–	Increased
Quality of arousal	Poor	Good
Addiction potential	Yes	No
Side effects	Yes	Rare
DNA/proteins synthesis	Decreased	Increased
NPY mediated activation of Hsp70	–	Increased

*Note*: Updated from References [27, 152].

**Figure 5:**
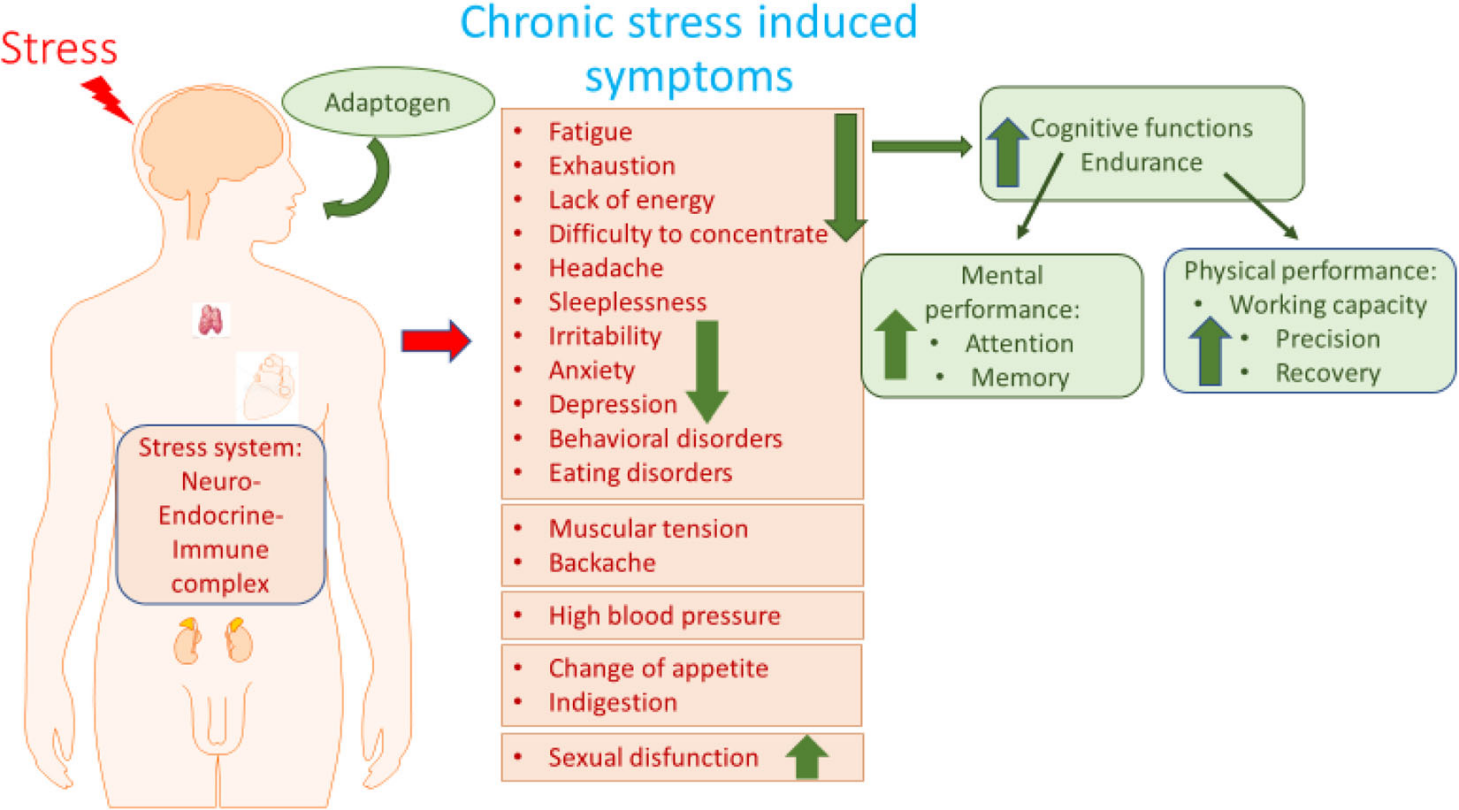
Chronic stress‐induced symptoms and effect of adaptogens, updated from authors’ drawings^14^ [Color figure can be viewed at wileyonlinelibrary.com]

Primarily, adaptogens have potential benefits in cases of behavior‐related disorders, mental illness, stress‐induced fatigue (Figure 5), and cognitive function.^11^, ^14^, ^15^, ^26^, ^27^, ^48^, ^74^, ^75^, ^216^, ^275^, ^350^, ^353^, ^354^, ^355^, ^356^, ^357^, ^358^, ^359^, ^360^, ^361^, ^362^, ^363^, ^364^, ^365^, ^366^, ^367^ In a number of clinical studies, the beneficial effects of adaptogens have been demonstrated on healthy subjects in stress conditions.^26^, ^27^, ^48^, ^74^, ^75^, ^324^, ^353^, ^356^, ^357^, ^359^, ^362^ This is especially true of the mental and physical performance of fatigue and mental strain. Furthermore, the efficacy of adaptogens in mild and moderate depression has been demonstrated.^275^, ^355^, ^358^, ^360^, ^363^, ^366^

The prophylactic use of adaptogens seems to be justified in healthy subjects for preventing aging‐related diseases, and to attenuate stress‐induced harmful effects.^26^, ^27^, ^95^, ^317^, ^368^, ^369^, ^370^, ^371^

Several systematic reviews and assessment reports have been conducted on the clinical efficacy and safety of ginseng,^2^, ^372^, ^373^, ^374^ *Eleutherococcus*,^375^ *Rhodiola*,^376^, ^377^, ^378^, ^379^, ^380^, ^381^, ^382^ *Withania*,^383^, ^384^, ^385^, ^386^, ^387^, ^388^ and other adaptogens on several indications such as cognitive function,^33^, ^72^ cardiovascular diseases,^389^ chronic pulmonary disease,^390^ prevention of common cold,^391^ and erectile dysfunction.^392^ The clinical evidence of the benefits of *W. somnifera* in male infertility is also promising but very limited to provide sufficiently robust evidence because of the small number of eligible studies and available data.^393^ The results suggest the potential role of *W. somnifera* in managing diabetes mellitus, but evidence is not robust because of insufficient available clinical data. Furthermore, well‐designed randomized controlled trials (RCTs) with a larger sample size and longer duration are warranted to evaluate its effect primarily on blood glucose, HbA1c, and insulin.^386^ In five studies conducted in patients with anxiety and stress, significant (in most cases) improvements were observed with *Withania* intervention as compared with placebo, but cases of potential bias were identified.^383^ There is some evidence from randomized, placebo‐controlled, double‐blind trials regarding the benefits of *W. somnifera* on cognitive function, such as improved performance on cognitive tasks, attention, and reaction time.^385^ However, the study population was heterogeneous, including older adults with mild cognitive impairment and adults with schizophrenia, schizoaffective disorder, or bipolar disorder.

In most of the early clinical studies on *Eleutherococcus* preparations conducted in the USSR in the 1960s and 1970s, positive results were commonly reported.^394^ However, most of these trials lacked good methodology (e.g., lack of randomization, proper control, blinding, statistical tools, description of inclusion and exclusion criteria, description of the medication, diagnosis, study design, and small sample size). In 2009, Li et al. assessed the efficacy and safety of *Eleutherococcus* in patients with acute ischemic stroke in a Cochrane systematic review. The authors included 13 RCTs (962 participants). The primary outcome measure in all included trials was the improvement of the neurological deficit after treatment. *Eleutherococcus* was found to significantly increase the number of participants with improvement in neurological impairment. However, because the risk of bias in all of the included trials was high, the authors concluded that much larger trials of greater methodological quality are required.^375^ In the EMEA assessment report dated March 25, 2014, the authors concluded that despite the large number of studies on the topic, *Eleutherococcus* root preparations do not reach the level of “well‐established use” scientific evidence sufficient to grant a marketing authorization, although in total, the data available are sufficient to justify further research on the concept of adaptogens.^3^

Similar decisions were made in 2011 and 2012 regarding Rhodiola^1^ and ginseng.^2^ The beneficial effects of ginseng on cognitive function have been demonstrated in several studies, but the evidence was not sufficient to achieve the designation of well‐established use in 2012 because of the heterogeneity of the investigated preparations, limited numbers of participants, differences in study design, and methodological quality.^2^ Because the number of clinical trials on the clinical efficacy of *R. rosea* was limited, we could not conclude that there was sufficient evidence for well‐established use in the treatment of fatigue or mental weakness. However, the data support the plausibility of the use of the traditional herbal medicinal products of *R. rosea* as adaptogens.^1^

In Sweden, Norway, and Denmark, *Rhodiola* traditional herbal medicinal product is indicated as an adaptogen in situations of decreased performance such as fatigue and sensation of weakness.

In a systematic review and meta‐analysis of 11 RCTs of *R. rosea*, Hung et al.^381^ concluded that “the methodological quality of most trials was moderate or good. Five of the 11 RCTs reached more than 3 points on the Jadad score (i.e., good quality). *R. rosea* may have beneficial effects on physical performance, mental performance, and certain mental health conditions. Only a few mild adverse events were reported. There is, however, a lack of independent replications of the single different studies”.

Extracts of Red Korean Ginseng have been tested extensively in mice and isolated cells infected with influenza virus. The antiviral protective effects were observed regardless of influenza virus strains, including various subtypes of H1N1, H3N2, H5N1, and H7N9. Mice inoculated with a lethal dose of virus and ginseng preparations were protected against weight loss with 100% survival rates during primary infection, and they developed immunity against secondary viral infection.^395^, ^396^ The use of various ginseng extracts to treat mice infected with influenza virus decreased the interleukin (IL)‐6 and IL‐8 cytokines and increased antiviral cytokine interferon (IFN) upon influenza virus infection.^397^, ^398^, ^399^, ^400^ It was demonstrated that ginsenosides, particularly Rb1, interact with viral hemagglutinin proteins, preventing the virus from binding to host cells and viral entry into the cytoplasm.^401^ Meanwhile, ginseng polysaccharide fraction exhibits a strong antiviral effect in mice infected with influenza A virus, predominantly by reducing the accumulation of tumor necrosis factor α (TNF‐α)/inducible nitric oxide synthase (iNOS)‐producing dendritic cells (tipDCs) in mouse lungs.^402^ Clinical trials suggest that ginseng is an effective prophylactic agent for respiratory infections, reducing the risk and duration of colds and flu and providing symptom relief.^403^, ^404^, ^405^

The efficacy and safety of *Andrographis*‐containing preparations were studied in patients with common cold in Scandinavia, South America, and India.^406^, ^407^, ^408^, ^409^, ^410^, ^411^ Evidence from a meta‐analysis of the results of 33 RCTs showed that *Andrographis* relieves inflammatory symptoms and shortens the duration of cough, sore throat, and sick leave/time to resolution when compared with usual care.^411^

Several epidemiological studies conducted in the USSR during the 1970s appeared to establish that *Eleutherococcus* root extract, given prophylactically, can reduce morbidity rates during an influenza virus epidemic as well as typical complications of influenza infection, such as bronchitis, pneumonia, and otitis.^3^ *Eleutherococcus* is an effective antiviral agent that induces IFN‐γ production^412^, ^413^, ^414^, ^415^, ^416^ and increases leukocyte, cytotoxic T‐cell, T‐helper, and B‐ and T‐lymphocyte counts in peripheral blood.^412^, ^417^, ^418^, ^419^, ^420^ The efficacy of adaptogens in the treatment of acute respiratory tract diseases is possibly also partially associated with the downregulation of proinflammatory NF‐kB signaling in various cells and tissues involved in the acute inflammatory response.

The fixed combination (Kan Jang) of *Andrographis* and *Eleutherococcus* has been used since 1979 in Sweden as an herbal medicine (“naturmedel”), with well‐established use (“naturläkemedel”) in Denmark since 1997 for reducing the severity and duration of symptoms of common cold.^3^ This combination was tested in controlled clinical trials for the treatment of common cold and influenza‐associated uncomplicated upper respiratory infections as well as for the prevention of common colds.^421^, ^422^, ^423^, ^424^ The studies confirmed the safety and superior efficacy of this combination regimen as compared with monodrug therapy,^425^ presumably because of its antiviral effects,^426^, ^427^, ^428^, ^429^, ^430^, ^431^, ^432^ effects on innate and adaptive immunity,^433^, ^434^, ^435^, ^436^, ^437^ and anti‐inflammatory, antioxidant, and detoxifying effects^438^, ^439^, ^440^, ^441^ of both adaptogenic plants as well as due to their synery.^25^ It should be noted that the postmarketing pharmacovigilance assessment of Kan Jang showed a high benefit–risk ratio: one adverse event in about 100,000 patients was recorded for the 23‐year period from the adverse event reports (concerning mainly allergic reactions) to the Swedish and Danish medical product agencies. Further studies are needed to evaluate the efficacy of these plants in patients with COVID‐19 and other viral respiratory invidious diseases.

One more possible benefit of adaptogens in respiratory tract infectious diseases might be their beneficial effect during patient convalescence. Adjuvant therapy with Chisan/ADAPT‐232, a fixed combination of *Eleutherococcus*, *R. rosea*, and *S. chinensis*, in pneumonia has a positive effect on patient recovery by decreasing the duration of the acute phase of the illness, increasing patient mental performance during the rehabilitation period, and improving patient quality of life (QOL).^354^ Both the clinical and laboratory results of the present study suggest that Chisan (ADAPT‐232) can be recommended in the standard treatment of patients with acute nonspecific pneumonia as an adjuvant to increase patient QOL and to expedite their recovery.

Dietary supplements containing *Rhodiola, Withania, Ginseng, Eleutherococcus, Schisandra*, and other adaptogenic plant extracts are widely used all over the world,^21^, ^69^, ^87^, ^160^, ^161^, ^261^, ^318^, ^442^, ^443^, ^444^, ^445^, ^446^ while in China, Korea, Japan, Russia, and some neighbor countries various pharmaceutical forms of adaptogenic plants form a part of official medicine.^447^, ^448^, ^449^ Overall, it is well documented now that adaptogens act polyvalently with positive effects on aging‐related disorders including atherosclerosis and other chronic inflammatory diseases, metabolic diseases, neurodegenerative cognitive impairment as well as cancer.^1^, ^2^, ^3^, ^4^, ^10^, ^13^, ^15^, ^17^, ^21^, ^44^, ^57^, ^69^, ^278^, ^444^ For example, numerous in vivo and in vitro studies on *P. ginseng* have shown its beneficial effects in aging, CNS disorders, and neurodegenerative and cardiovascular diseases, cancer, immune deficiency, and hepatotoxicity. Clinical trials have been conducted on the effects of ginseng preparations on cognitive function, lipid and glucose metabolism, cardiovascular function, erectile dysfunction, quality of life, improvement of the immune system, and chronic respiratory diseases.^57^ All of them are associated with the metabolic regulation of homeostasis and threatened adaptability of the stress system. Adaptogenic plants possess compounds that exhibit anticancer activity and potentiate the effects of antitumor drugs, suggesting that they can be used alone or as adjuvants to conventional chemotherapy to improve their efficacy or reduce radiotherapy‐ or chemotherapy‐induced toxicity,^348^ for example, nausea and vomiting.^114^, ^450^ Supplementation with adaptogens is also considered a promising therapy for cancer‐related fatigue, a debilitating syndrome that persists for years in many cancer survivors.^88^

More evidence from controlled clinical studies supporting health claims and indications for use in diseases are required.

## 5 CORE RATIONAL OF THE ADAPTOGENIC CONCEPT

### 5.1 Mechanisms of adaptogenic and stress‐protective actions

The pathogenesis of complex diseases as well as the adaptive stress response, inflammation, and senescence are multistep processes which involve extracellular and intracellular communications at differing stages of stress regulation and cannot be limited to the few biochemical interactions that occur in the brain or other tissues. Clearly, for the description of the mechanism of action of adaptogens the reductionist model that assumes a single drug—single receptor interaction is insufficient and not valid. Adaptogens have many molecular targets^16^, ^17^ involved in the metabolic regulation of homeostasis at both the cellular and systemic levels and play a role of stress response modifiers.^11^, ^16^, ^17^, ^19^, ^20^, ^21^, ^22^, ^23^, ^24^, ^25^, ^26^, ^27^, ^28^, ^49^, ^56^, ^60^

Network pharmacology with the use of the systems biology offers exciting new opportunities for understanding such complex systems.^16^ During the past several decades, many molecules, signaling pathways, and networks targeted by adaptogens have been identified.^11^, ^16^, ^18^, ^19^, ^20^, ^21^, ^22^, ^23^, ^24^, ^25^, ^26^, ^27^, ^28^, ^49^, ^56^, ^60^ They include stress hormones and some other important mediators of homeostasis regulation such as the molecular chaperons Hsp70, neuropeptide Y, G protein‐coupled receptors (GPCRs), dopamine‐cAMP‐PKA‐CERT, IP3, PLC, DAG, phosphoinositide 3‐kinase (PI3K), nuclear factor kappa‐light‐chain‐enhancer of activated B cells (NF‐κB)‐mediated signaling pathways, stress‐activated kinase c‐Jun N‐terminal kinase (JNK), forkhead box protein O3 (FOXO3), cortisol, estrogens, and nitric oxide (NO).^11^, ^16^, ^18^, ^19^, ^20^, ^21^, ^22^, ^23^, ^24^, ^25^, ^26^, ^27^, ^28^, ^49^, ^56^, ^60^ The mechanisms of action of adaptogens are mainly associated with metabolic regulation via extracellular communication of hypothalamic–pituitary–adrenal (HPA)‐axis hormones and activation of intracellular adaptive stress response signaling pathways.^16^

#### 5.1.1 Effect of adaptogens on extracellular communications within the neuroendocrine‐immune system

**Figure 6:**
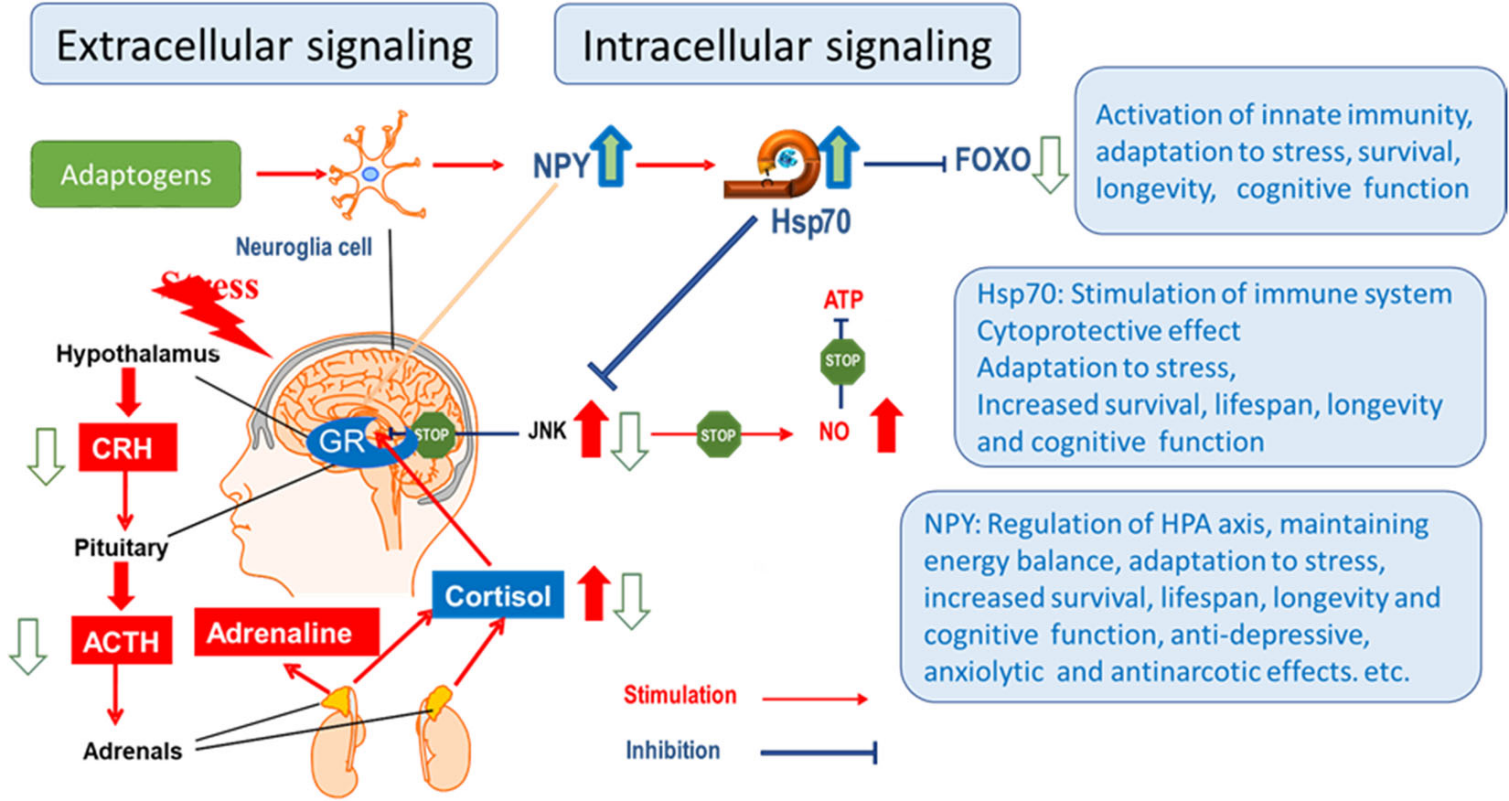
Hypothetical mechanism of action underlying the effects of adaptogens on the adaptive stress response in the hypothalamic–pituitary–adrenal axis: forkhead box O, neuropeptide‐Y (NPY), and Hsp70 signaling. Persistent chronic stress induces and blockage of negative feedback regulation of cortisol and disruption of ATP synthesis. During stress, corticotropin‐releasing hormone (CRH) is released from the hypothalamus, followed by the release of adrenocorticotropic hormone (ACTH) from the pituitary, which stimulates the release of adrenal hormones and NPY. Feedback regulation of overreaction is triggered by cortisol release from the adrenal cortex, followed by binding to glucocorticoid receptors (GRs) in the brain, which halts the further release of brain hormones, resulting in decreases of cortisol to normal levels. Although mild stress (eustress) is a vital part of life, chronic and severe stress can cause depression associated with the production of active oxygen‐containing molecules including nitric oxide, which inhibits ATP formation. The stress‐induced signaling protein c‐Jun N‐terminal kinase (JNK) inhibits GRs. Subsequently, this feedback control is inhibited and the cortisol content in the bloodstream of depressed patients is permanently high, which is associated with impaired memory, decreased ability to concentrate, fatigue, among others. Adaptogens normalize increased cortisol/corticosterone levels in the bloodstream and saliva of humans or animals^12^, ^357^ presumably due to direct interaction with GRs. Adaptogens also attenuate elevated JNK and cortisol levels during stress and activate the generation of Hsp70, which inhibits JNK. Therefore, the nitric oxide level no longer rises, and ATP production is not inhibited (adapted from authors’ drawings^26^) [Color figure can be viewed at wileyonlinelibrary.com]

The hypothetical mechanism of adaptogens’ action on HPA‐axis hormones in stress is presented in Figure 6. The HPA axis plays a pivotal role in regulating the majority of endocrine hormones associated with the CNS. Stress hormones regulate growth, appetite, blood pressure, emotion, sexual function, body temperature, sleep, biorhythms, and hydration. They are produced by the endocrine system, are secreted into the bloodstream, and target other tissues to regulate physiological functions. The main function of stress hormones is to maintain homeostasis to counteract stress

Ginsenoside Rg1 directly interacts with glucocorticoid receptor (GR) ligand‐binding sites and behaves as a partial agonist of GR. Ginsenoside Rb1 is a functional ligand of the estrogen receptor (ER).

Along with CRH, another primary upstream mediator of extracellular communications stimulated by adaptogens is the stress hormone neuropeptide‐Y (NPY).^23^, ^28^ Stimulation and release of NPY into the blood circulatory system are innate defence responses to mild stressors (adaptogens), which increase resistance to stress. This leads to stress‐protective and adaptive effects via various elements of the endocrine, immune, central nervous, sympathetic, cardiovascular, and gastrointestinal systems. Both Hsp72 and NPY play essential roles in stress, and pathogenesis of aging‐related diseases. The antinarcotic effects of adaptogens are mediated by NPY, which is an important intermediate involved in morphine tolerance and opioid dependence.

#### 5.1.2 Molecular mechanisms of action—Effects on intracellular signaling pathways

Gene expression analysis has helped to gain an improved understanding of the molecular mechanisms of action of adaptogenic plants and elucidation of adaptive stress response signaling.^17^, ^23^, ^24^, ^25^, ^451^, ^452^ One recent study in which the gene expression profiles of isolated brain cells were exposed to adaptogens, showed that at least 88 of the 3516 genes regulated by adaptogens modulate many signaling pathways involved in the adaptive stress response.^17^ Genes encoding neurohormones, transmembrane channels, and receptors, transcription regulators and ligand‐dependent nuclear receptors, protein kinases phosphatases, peptidases, metabolic enzymes, chaperones and other intermediates of intra‐ and extracellular communications (Table 9) are key elements in several canonical pathways involved in defence response, survival, longevity, and in maintaining of cellular and organismal homeostasis.

**Table 9:** Genes regulated by adaptogens

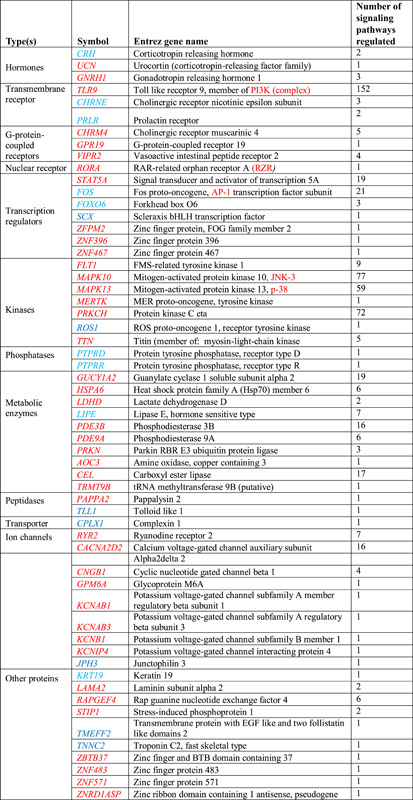			

*Note*: Upregulated genes are in red color while downregulated genes are in blue color text.

Some of these proteins play key roles in regulating numerous processes. As an example, all adaptogens upregulate TLR9, a member of the PI3K (complex) gene‐encoding transmembrane receptor that plays key roles in regulating 152 signaling pathways including glucocorticoid receptor signaling, interleukins IL‐2, IL‐3, IL‐4, IL‐6, IL‐7, IL‐8, IL‐9, ILK, IL‐12, IL‐15, IL‐17A, IL‐17 signaling, B‐ and T‐cell receptor signaling, leukocyte extravasation, extracellular signal‐regulated protein kinase (ERK)/MAPK, PI3K/AKT signaling in the pathogenesis of influenza, lipopolysaccharide‐stimulated MAPK signaling, p53 and JNK signaling, production of NO and reactive oxygen species (ROS) in macrophages signaling, eNOS signaling NO signaling in the cardiovascular system, leptin signaling in obesity, type II diabetes mellitus signaling, IGF‐1 and insulin receptor signaling, prolactin signaling, AMPK signaling, and so forth. ^17^

All adaptogens upregulate protein kinase C (PKC) eta, an enzyme encoded by the PRKCH gene that plays key roles in the regulation of 72 signaling pathways including CRH signaling, androgen signaling, prolactin signaling, growth hormone signaling, melatonin signaling, Gαq signaling, feedback in cAMP signaling, Nrf2‐mediated oxidative stress response, production of NO and ROS in macrophages, mTOR signaling, NF‐κB activation by viruses, calcium‐induced T lymphocyte apoptosis, protein kinase A signaling, phospholipase C signaling, eNOS signaling, opioid signaling pathway, neuropathic pain signaling in dorsal horn neurons, axonal guidance signaling, CREB signaling in neurons, dopamine‐DARPP32 endothelin‐1 signaling, α‐adrenergic signaling, nNOS signaling in neurons, synaptic long‐term potentiation and synaptic long‐term depression signaling pathways.^17^

All adaptogens upregulate mitogen‐activated protein kinases MAPK10 and MAPK13 which correspondingly involved in the regulation of 77 and 58 signaling pathways, including adaptive stress response signaling survival and longevity. These findings support the use of adaptogenic plants in TMS as a panacea for the treatment of numerous diseases.

All adaptogens tested (*R. rosea L., E. senticosus, W. somnifera, R. carthamoides, and B. alba*) activate the melatonin signaling pathway by acting through two GPCRs MT1 and MT2, and upregulating the ligand‐specific nuclear receptor RORA, which plays a role in different common aging diseases such as neurological disorders, hypertension, dyslipidemia, intellectual disability, retinopathy, and cancer. Furthermore, melatonin activates adaptive signaling pathways and upregulates the expression of *UCN, GNRH1, TLR9, GP1BA, PLXNA4, CHRM4, GPR19, VIPR2, RORA, STAT5A, ZFPM2, ZNF396, FLT1, MAPK10, MERTK, PRKCH*, and TTN, which are commonly regulated by all adaptogens tested.^17^

The common features of recently tested extracts (*B. alba L., Boswellia serrata Roxb. ex Colebr., Curcuma longa L., E. senticosus* (*Rupr. & Maxim*.) *Maxim, Rhaponticum carthamoides* (*Willd*.) *Iljin*, *R. rosea L., and W. somnifera* (*L*.) *Dunal*) are related to the downregulation of *ALOX12*, which is also associated with the neuroprotective action of these medicinal plants as well as their potential benefits in neurodegenerative diseases.^452^

**Figure 7:**
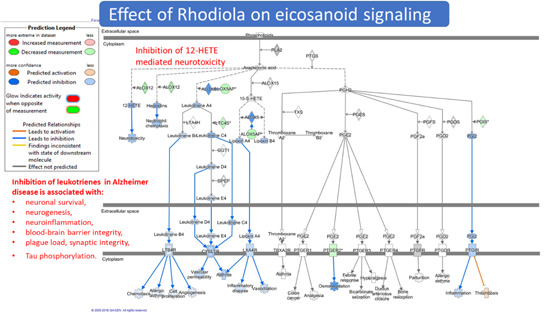
Effect of *Rhodiola* extract on the eicosanoids signaling pathway. Upregulated genes are shown in red color, while downregulated genes—in green color^452^ [Color figure can be viewed at wileyonlinelibrary.com]

*R. rosea, W. somnifera*, and *E. senticosus* downregulate the expression of key genes (*ALOX5AP, DPEP2, LTC4S*) involved in the biosynthesis of leukotrienes A, B, C, D, and E, resulting in inhibition of the leukotriene signaling pathway suggesting their potential benefits in Alzheimer's disease (Figure 7).^452^

Adaptogens exhibit multitarget node of action targeting several receptors including receptors for corticosteroid, mineralocorticoid, progestin, estrogen, serotonin, NMDA, nicotinic acetylcholine, receptor tyrosine kinases, and many GPCRs.^11^, ^16^, ^18^, ^19^, ^20^, ^21^, ^22^, ^23^, ^24^, ^25^, ^26^, ^27^, ^28^, ^44^, ^49^, ^56^, ^60^, ^453^, ^454^, ^455^, ^456^, ^457^, ^458^, ^459^, ^460^, ^461^, ^462^, ^463^, ^464^, ^465^, ^466^, ^467^, ^468^, ^469^, ^470^, ^471^, ^472^, ^473^ Numerous molecular network interactions (including feedback regulation of the neuroendocrine and immune systems) resulting in agonist‐dependent antagonism are presumably the most suitable model for understanding the mechanism of action of adaptogens.^16^

Interactive pathway analysis has demonstrated that adaptogens targets mediators of extracellular communications, intracellular networks, and signaling pathways, which are involved in stress‐induced and aging‐related disorders such as chronic inflammation, atherosclerosis, neurodegenerative cognitive impairment, metabolic disorders, and cancer.^17^, ^23^, ^24^, ^25^, ^368^ Importantly, the effect on every disease is multitargeted. As an example, *Robiola* regulates 22 genes, which are deregulated in mood disorders, including 14 genes that are deregulated in depression: *ADRA2B, AQP4, CACNB2, CCKBR, CHRNA1, CHRNB4, CHRNG, ESR1, GRIA3, GRIN1, KCNK2, MYOM1, NCAM1*, and *PDE11A*.^24^, ^275^

#### 5.1.3 Mechanisms underlying the cytoprotective, antioxidant, and antitoxic activities of adaptogens

Cytoprotective, antioxidant, and antitoxic effects of various adaptogenic preparations have been shown in many isolated cells as experimental models and (in vitro and ex vivo) in animals.^1^, ^2^, ^3^, ^4^, ^9^, ^26^, ^27^, ^69^, ^71^, ^474^ Extensive research on *E. senticosus* reveals its antitoxic, neuroprotective, hepatoprotective, cardioprotective, antioxidant, immunomodulating, and antiviral activities along with stress‐protective, antifatigue, hypoglycaemic, antidepressant and antiproliferative effects.^3^, ^8^, ^46^, ^474^, ^475^, ^476^, ^477^, ^478^, ^479^, ^480^, ^481^ As an example, *E. senticosus* inhibits the cadmium‐induced apoptosis and mitosis of hepatocytes in mice, and significantly decreased cadmium concentration in their liver and blood.^478^ It has been shown that hepatoprotective effect of *E. senticosus* extract is triggered by upregulation of expression Nrf2 and activation of innate antioxidant enzymes that increase the ratio of reduced versus oxidized glutathione in liver homogenate and serum.^226^ Repeated administration of *E. senticosus* preparation decreased isoproterenol induced cardiotoxicity and increased ventricular fibrillation threshold in rats with post‐infarction cardiosclerosis.^480^, ^481^ *Eleutherococcus* reduces the toxic effects of cytostatic drugs (cyclophosphane, etimidine, benzotef, sarcolisyne, ribomicine, 6‐mercaptopurine, dopane thiophosphamide, trichlortriethylamin) as chemotherapy including loss of body weight, increased mortality, decreased life span, reduction of tumor growth, thymic involution, haematopoiesis, and immunosupression. Adjuvant treatment with *Eleutherococcus* significantly increases the survival of rodents following etimidine treatment for carcinoma (100% survival vs. 70% control [etimidine]). Similarly, adjuvant treatment with *Eleutherococcus* significantly increases survival following thiophosphamide treatment (85% survival *vs*. 47% control [thiophosphamide]).^482^, ^483^, ^484^, ^485^

The antioxidant and hepatoprotective activity of *Eleutherococcus* and *Andrographis paniculate* preparations have been reviewed in EMA assessment report.^3^, ^4^ The chemopreventive effects of *A. paniculata* and Andrographolide against cyclophosphamide (CTX)‐induced urothelial toxicity were previously demonstrated.^486^ Both substantially lowered the elevated levels of IL‐2 and IFN‐γ and reduce CTX‐induced toxicity during CTX treatment.^486^ In another study, the aqueous extract of *A. paniculata* was shown to attenuate gentamicin‐induced nephrotoxicity decreasing blood levels of urea, creatinine, and urea nitrogen levels in rats.^487^

*A. paniculata* and andrographolide exhibit an extremely wide array of pharmacological activities^4^, ^425^, ^488^, ^489^, ^490^, ^491^, ^492^ including adaptogenic,^160^ antioxidant, chemopreventive,^4^ and neuroprotective activities.^361^, ^493^, ^494^, ^495^, ^496^

The cytoprotective effect of several adaptogenic plant extracts on chemotherapeutics‐induced dramatic impact on transcriptome‐wide RNA microarray profiles of neuroglia cells culture have been recently studied.^497^, ^498^, ^499^ The fixed combination 5‐fluorouracil, epirubicin, and cyclophosphamide (FEC) has been shown to deregulate 67 genes involved in the reduction of neuronal development, 37 genes involved in development of the sensory system, 12 genes involved in axon extension, and 3 genes involved in neuronal migration. Pretreatment of cells with *A. paniculata* prevented the FEC‐induced deregulation of genes involved in regulation of neuronal death, neurogenesis, and other vital functions in the nervous system. Similar cytoprotective effects exhibit a fixed combination of *A. paniculate* with *E. senticosis*, which prevented the FEC‐induced deregulation of gene expression involved in migration of T98G neuroglia cells, axon extension, conduction of nerves, and other neuronal functions associated with cognitive impairments. Adaptogens significantly modify FEC‐induced deregulation of genes involved in the regulation of cell morphology, synaptic, mitochondrial function, and protein‐related functions suggesting their potential neuroprotective and hepatoprotective effects, which are associated with FEC‐induced adverse events in cancer chemotherapy. The authors concluded that adjuvant treatment with adaptogens can prevent mild cognitive impairments and “chemobrain” effect associated with cancer chemotherapy.^497^ It is noteworthy that adaptogens potentiate the cytotoxic effects of chemotherapeutics in human T98G glioblastoma cells.^498^

There are several mechanisms underlying the cytoprotective and antitoxic effects of adaptogens.

**Figure 8:**
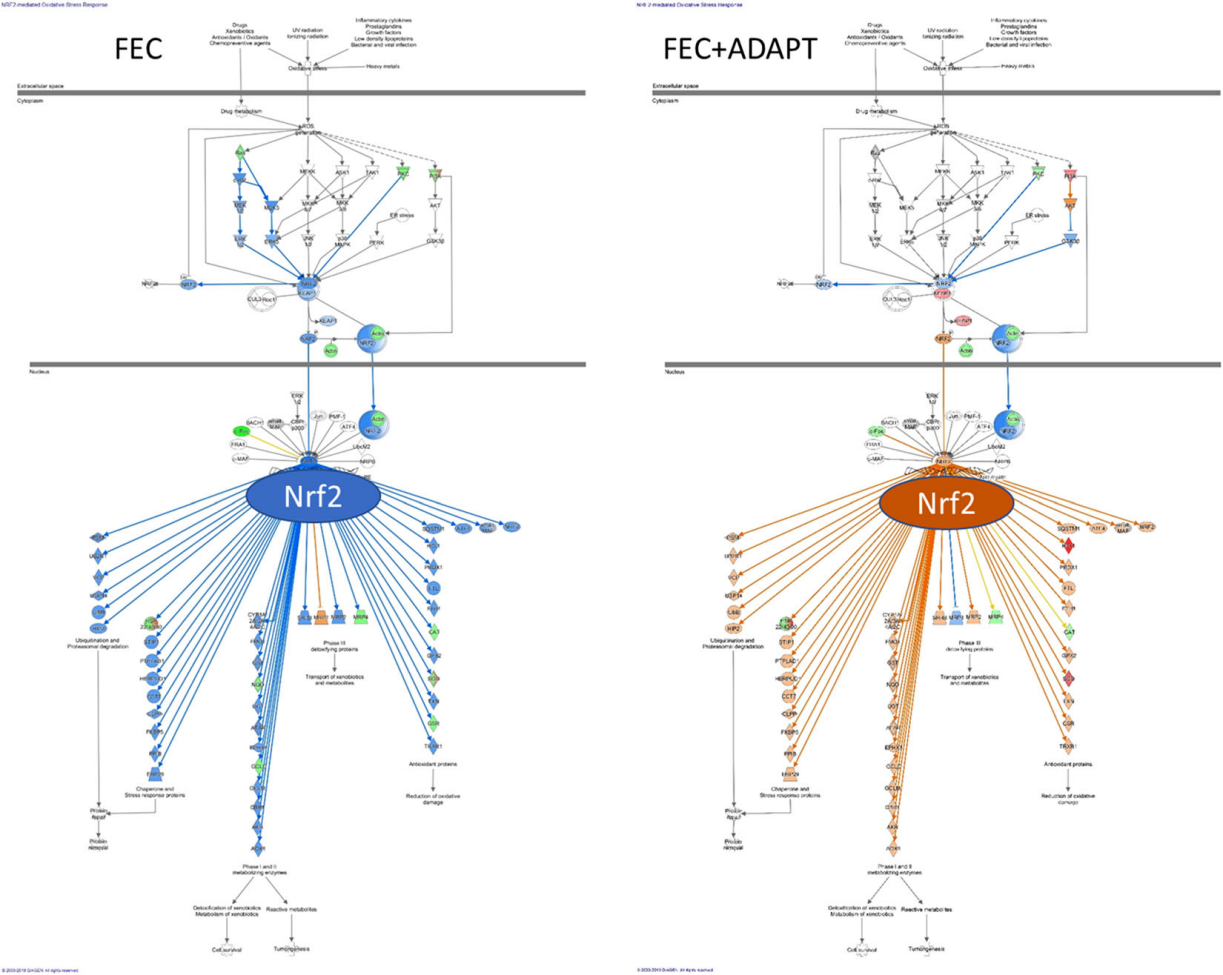
Adaptogens exhibit antioxidant and detoxifying effects presumably by activation of the Nrf2/ARE pathway. Nrf2 is a principal regulator of redox homeostasis normally retained in the cytoplasm by association Kelch‐like ECH‐associated protein‐1 (Keap1). Upon exposure of cells to oxidative stress, Nrf2 is phosphorylated in response to PKC, phosphatidylinositol 3‐kinase (PI3K), and MAPK pathways. After phosphorylation, this complex dissociates and Nrf2 translocates to the nucleus where it binds to the ARE and triggers expression of antioxidant and detoxifying genes including superoxide dismutase, glutathione S‐transferase, NAD(P)H quinone oxidoreductase 1, and heme oxygenase 1. Thus, activation of Nrf2 translocation or upregulation of gene expression resulting in activation of the Nrf2 signaling pathway is the key mechanism of the cellular defense response^500^, ^501^ associated with the antioxidant effects of medicinal plants, and particularly of adaptogenic plants, which are useful in stress‐ and aging‐related diseases [Color figure can be viewed at wileyonlinelibrary.com]

One of them is the Nrf2/antioxidant response element (ARE) signaling pathway, which a key defense response signaling pathway regulating the expression of phase II detoxifying enzymes in response to toxic stimuli (Figure 8).

An imbalance between the production of reactive oxygen radicals and their degradation results in oxidative stress. Reactive intermediates interact with polyunsaturated fatty acids, proteins, and RNA and DNA fragments, initiating numerous redox reactions that damage many cellular components such as the membrane, mitochondria, and nucleus, which leads to dysfunction of cellular processes and homeostasis, and triggers apoptosis and necrosis. Oxidative stress is increased in chronic inflammation and aging‐related disorders including atherosclerosis, angiogenesis, and neurodegeneration.^502^ The feedback cellular response is associated with activation of defence mechanisms including induction of antioxidant and detoxifying enzymes and molecular chaperones. Several adaptive signaling pathways such as p38, PKC, ERK, JNK, and PI3K signaling may activate Nrf2. Two other adaptive signaling pathways involving NF‐κB and FOXO transcription factors are important in neuronal stress adaptation.^503^, ^504^, ^505^, ^506^

Although adaptogens at high concentrations are potent radical scavengers, in lower amounts, they may activate some intracellular adaptive stress response signaling pathways resulting in the expression of cytoprotective proteins including neurotrophic factors, protein chaperones, antioxidant and phase II enzymes, and antiapoptotic proteins. One of them is transcription factor Nrf2.^151^

**Figure 9:**
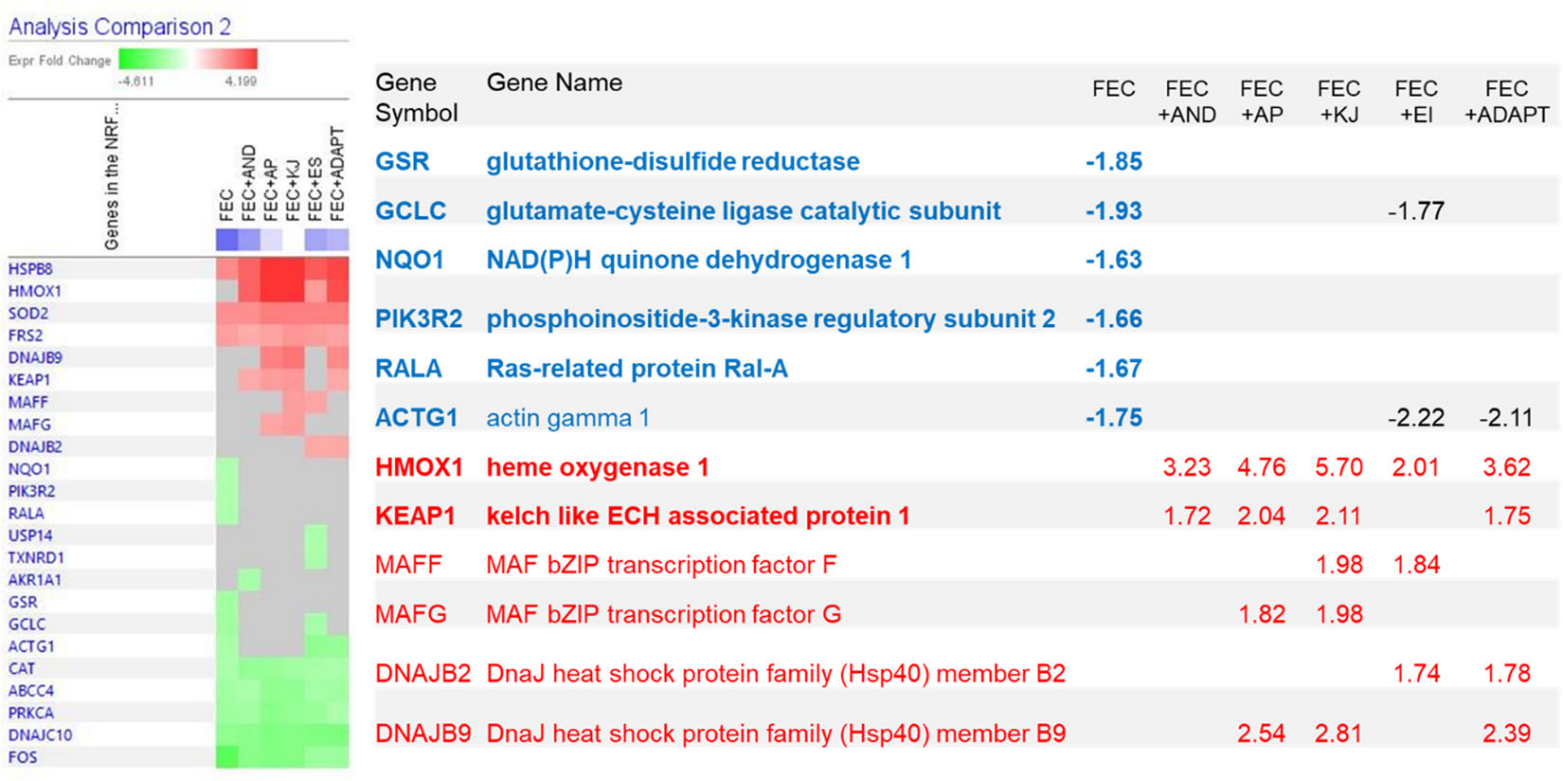
Adaptogens prevents the chemotherapy (FEC)‐induced downregulation of genes activating production of antioxidant and detoxifying proteins and upregulates genes involved in reduction of oxidation damage via Nrf2 signaling. Upregulated genes are shown in red, whereas downregulated genes are shown in blue [Color figure can be viewed at wileyonlinelibrary.com]

The beneficial effects of adaptogens appear to be related, at least in part, to their ability to activate the Nrf2/ARE pathway (Figure 8) and regulate the number of genes playing important roles in activation of the production of antioxidant and detoxifying proteins and genes involved in the reduction of oxidation damage (Figure 9).

**Figure 10:**
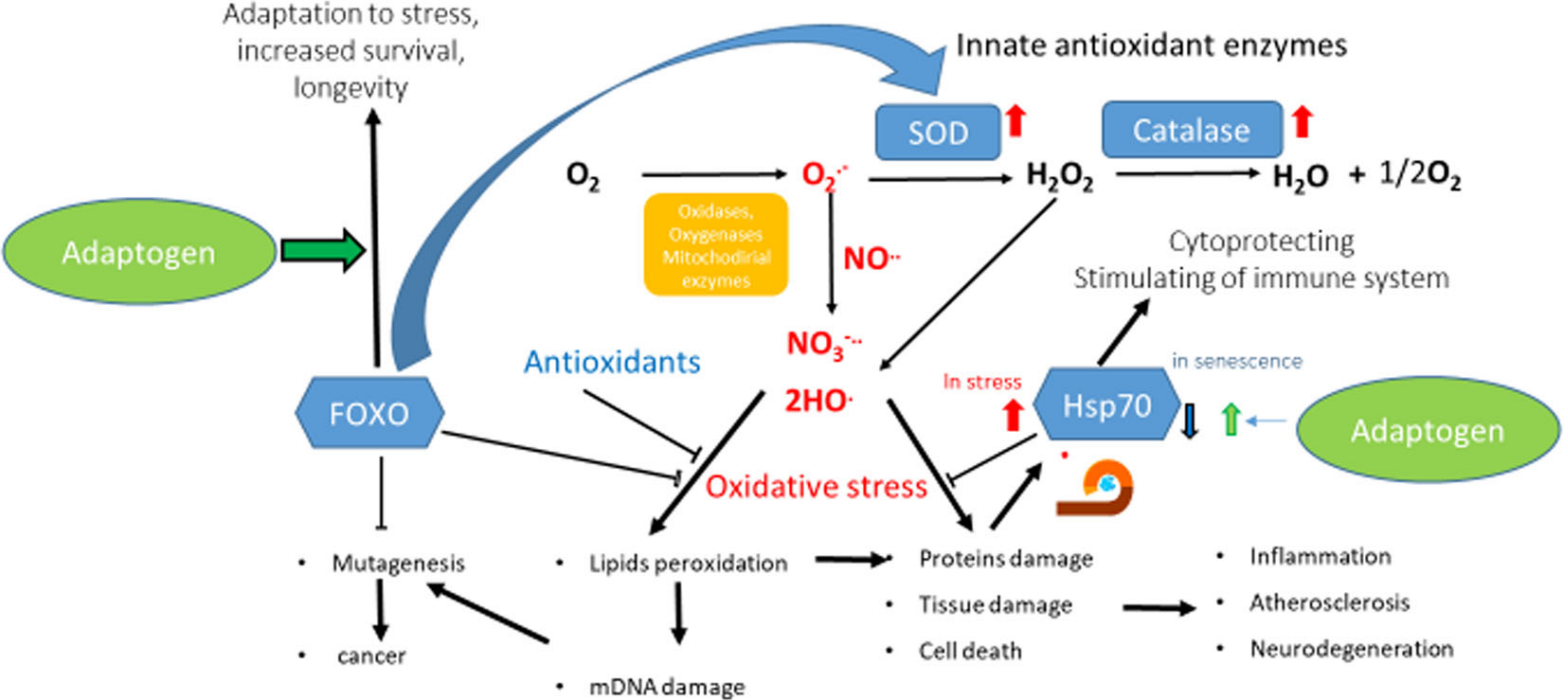
Hypothetical mechanism of action of adaptogens in the regulation of the innate antioxidant system and oxidative stress‐induced apoptosis in aging. According to the free radical theory of aging, the organisms are continuously exposed to reactive oxygen species containing molecules/species (ROS), which are produced as by‐products of normal cellular metabolism. When the innate antioxidant system (glutathione peroxidase, superoxide dismutase, and catalase) incompletely deactivates ROS, increasing cellular oxidative damage induces irreversible functional changes leading to early senescence and to aging‐associated diseases. Oxidative stress triggers many signaling pathways, including FOXO and Hsp70 mediated pathways. Adaptogens upregulate Hsp70, which directly regulates FOXO signaling and promote translocation of FOXO/DAF‐16 to nucleus triggering activation antioxidant systems and antiaging programs. Updated from authors’ drawings^16^ [Color figure can be viewed at wileyonlinelibrary.com]

Other possible cytoprotecting mechanisms of adaptogens related to drug toxicity, oxidative stress, chronic inflammation, and aging‐related disorders include their effects on Hsp70 and FOXO expression (Figure 10).

#### 5.1.4 Aging‐associated disorders

Aging‐associated disorders arise from declining capabilities to cope with stress, to sustain cellular and system homeostasis, and to maintain physiological functions. These disorders are associated with neurodegeneration (common for Alzheimer's disease, Parkinson's disease, and senile dementia), atherosclerosis (cause of cardiovascular and cerebrovascular diseases), immune regulation (dysregulated in cancer, autoimmune, and chronic inflammatory diseases), and endocrine/metabolic dysfunction (imbalanced in diabetes and obesity).

Overproduction of ROS in stress‐induced condition leads to destruction of proteins, including those triggering genetic programs of cellular senescence and cell death (apoptosis). Attenuation of functions, increasing damage to proteins, and toxic protein aggregates initiate aging‐related changes leading to disease, senescence, and reduced life span. In aging cells, substantially decreased expression of heat shock protein Hsp70 and its precursor, heat shock transcription factor HSF1, correlates with a decreased ability to cope with stress.^507^, ^508^ When cells are exposed to stress resulting in protein damage, HSF1 initiates the production of molecular chaperone Hsp70,^508^ which repairs proteins by folding denatured parts of proteins and promotes the degradation of irreversibly damaged proteins and their aggregates. In addition, Hsp70 directly protects cells against switch to apoptosis. Decreased expression of HSF1 and Hsp70 in brain cells is observed in Alzheimer's disease.^509^, ^510^ It is associated with the accumulation of protein aggregates of β‐amyloid peptide and cytoskeletal protein.^511^ Aging‐related decline of hepatic Hsp70 expression results in decreased liver detoxification^512^ and protection from toxic substances.^513^ Decrease of Hsp70 is coupled with upregulation of stress‐activated protein kinase (JNK) dependent apoptosis and progression of cancer Stress‐induced decline in induction of Hsp70 observed in humans, is associated with aging and aging‐related disease.^514^ Amazingly, in some individuals which are more than 100 years old, Hsp70 does not decrease with age.^515^

**Figure 11:**
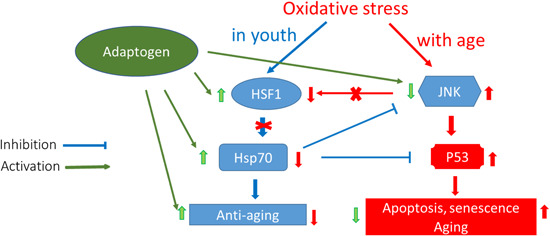
Effects of age and adaptogens on longevity regulatory pathways during oxidative stress. HSF1, heat shock factor 1; Hsp70, heat shock protein 70; JNK, JN kinase; P‐53, p‐53 transcription factor;**↓** or **←, **for activation; **x**, for blocking; |, for inhibition; bold text for the prevailing process [Color figure can be viewed at wileyonlinelibrary.com]

In young age, the balance between pro‐ and antiaging JNK‐mediated programs is shifted in favor of Hsp70 (Figure 11). Apparently, oxidative stress does not affect survival and reproduction of young cells because stress‐activated Hsp70 blocks JNK‐stimulated apoptosis. Enhanced levels of Hsp70 correlate with increased life span. In contrast, with age, when induction of Hsp70 is reduced, the balance shifts in favor of the aging and apoptosis programs. Consequently, even weak oxidative stress can induce the degeneration of neuronal cells and the progression of aging‐related diseases. The ability to respond effectively to stress by generating increased Hsp70 correlates with high adaptability and increased life span.^516^ Thus, the onset of neurodegenerative diseases and other aging‐related illnesses may be delayed by modulating these two pathways.^517^

The adaptogens *R. rosea*, *S. chinensis*, and *E. senticossus*, alone and in combination, up‐regulate transcription factor HSF1, and increase generation of molecular chaperon Hsp70 in vitro and in vivo.^19^, ^20^, ^21^, ^28^, ^42^, ^518^, ^519^, ^520^, ^521^ Adaptogens also inhibit stress‐activated protein kinase JNK,^18^ a key mediator of apoptosis and aging (Figure 11). Furthermore, adaptogens trigger translocation of transcription factor DAF‐16 (FOXO) from the cytoplasm into the nucleus.^95^ The protective effect against myocardial ischemia‐reperfusion injury via increase of Hsp25 and Hsp70 expression in rat hearts was described for schizandrin B, an active constituent of *S. chinensis*.^518^ Induction of Hsp27 and Hsp70 genes and protein expression were observed in a dose‐dependent manner after oral administration of schizandrin B to rats.^520^

The prolongation of life span and increased survival under stress after treatment with preparations from *R*. *rosea, S. chinensis, E. senticossus, W. somnifera*, and *P. ginseng* have been shown. in the fruit fly *Drosophila melanogaster*,^370^, ^371^, ^522^, ^523^ the nematode *Caenorhabditis elegans*,^95^, ^317^, ^524^ and yeast *Saccharomyces cerevisiae*.^525^ Oral supplementation with salidroside or extracts of *E. senticosus, S. chinensis*, and *R. rosea* significantly decreased stress‐induced elevation of p‐SAPK/p‐JNK in rabbits subjected to restraint stress.^18^ Based on these observations, it was suggested that adaptogens acts as mild stressors inducing enhanced stress resistance and an extended life span.^368^

Adaptogens regulate G‐protein signaling phosphatidylinositol and phospholipase C pathways (Figure 4). *R. rosea, S. chinensis, and E. senticossus* up‐regulate the expression of *PLCB1* gene, which encodes phosphoinositide‐specific phospholipase C (PLC), and the *PI3KC2G* gene, which encodes PI3Ks.^23^ G‐protein activated, phospholipase C (PLC) catalyzes the hydrolysis of phosphatidylinositol 4,5‐bisphosphate (PIP2) into diacylglycerol (DAG) and inositol‐1,4,5‐triphosphate (IP_3_) that is involved in numerous intracellular signaling pathways associated with various diseases including depression and cancer. DAG triggers protein kinase C (PKC), which phosphorylates numerous proteins and plays an important role in tumor progression. PI3K is a key upstream mediator of intracellular signaling related to regulation of NF‐kB‐mediated defence responses and apoptosis as well as to long‐term potentiation of neurotransmission, which improves memory and learning.^526^, ^527^

*R. rosea, S. chinensis, and E. senticossus* downregulate the *CETP* gene ^23^ expression, which regulate the biosynthesis of cholesteryl ester transfer protein that facilitates the transport of cholesterol esters and triglycerides between low‐density lipoproteins (LDL) and high‐density lipoproteins (HDL).^528^ Inhibiting of expression of *CETP* may be helpful in the treatment of atherosclerosis, cardiovascular and metabolic diseases.^529^

Adaptogens downregulate the *ESR1* gene.^23^ *ESR1* encodes estrogen receptor α (ERα), which is overexpressed in some cancers.^530^, ^531^, ^532^ Downregulation of expression of *ESR1* by Rhodiola and other adaptogens may be effective for preventing and treating some aging‐related cancers such as breast cancer, ovarian, colon, prostate, and endometrial cancers.^23^, ^24^ The neuroprotective effects of adaptogens^533^, ^534^, ^535^, ^536^, ^537^, ^538^, ^539^, ^540^, ^541^, ^542^ may be also partially associated with the upregulation of ESR1 in glia cells, as estrogen signaling through ERα decreases the inflammatory neurodegeneration via its effect on astrocytes. Since pretreatment with adaptogens is known to adapt the cell to stress^12^, ^95^, ^351^ it is possible that the adaptogens‐mediated downregulation of ERα gene expression signals the glia cells to initiate feedback regulation of ERα. This concept is usually associated with inflammation, a protective reaction to infection (‘turn on’ defense system). A feedback mechanism that downregulates pathogen‐induced inflammatory response is triggered (e.g., increased secretion of cortisol and release of anti‐inflammatory cytokines) to prevent an overreaction (“turn off” defence system). Because mild stress is generally a protective reaction to activate innate immunity, in this context, adaptogens initiate stimulation of the innate defence system, including ERα, as one element of the stress system.

Along with canonical mode of steroid receptor action related to regulation of transcription of target genes in the nucleus, estrogen activated membrane bound CRα triggers PI3K/PLC signaling pathways in brain cells to modulate neuronal function and apoptosis.^434^, ^444^ Estrogen treatment attenuates transcription at estrogen response elements sites in glioma cells.^444^ Since Rhodiola upregulates PLCB1, PI3KC2G, and c‐AMP related genes, and modulates NO and JNK it was suggested that Rhodiola and estrogens interfere with each other in some way.^340^ While both are neuroprotective, it is remaining unclear whether they are mimetic and in competition or are naturally antagonistic.

**Figure 12:**
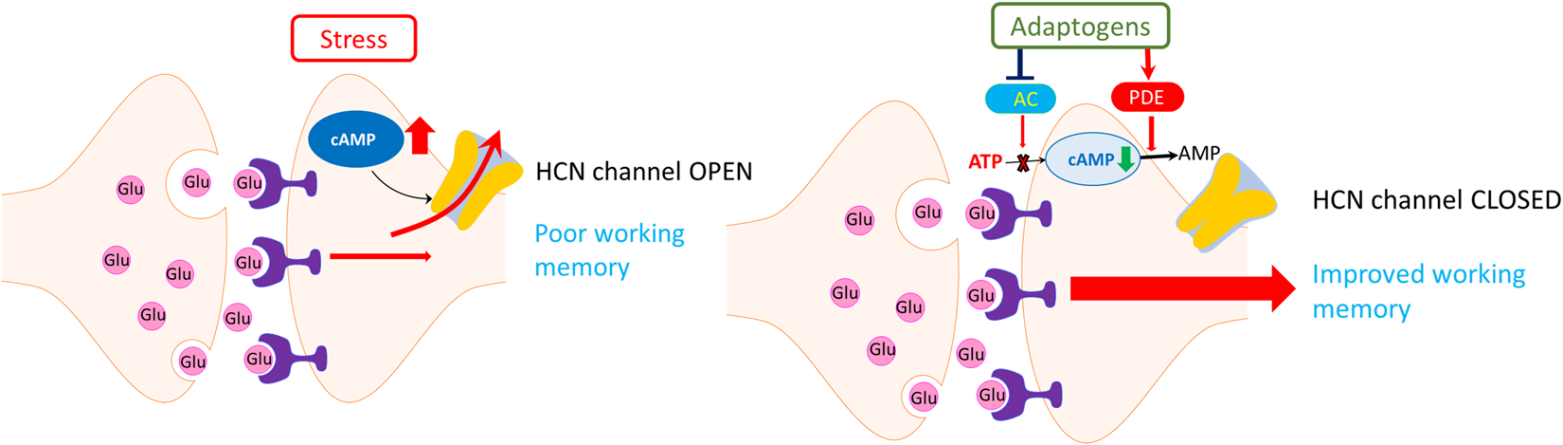
Effect on cAMP‐mediated signaling in prefrontal cortex neurons. HCN channel opening shunts synaptic inputs onto dendritic spines and reduces strength of prefrontal cortex network activity. cAMP opens HCN channel that decreases the efficacy of cortical inputs. Adaptogens downregulate AC and upregulate PDE that decreases cAMP level in brain cells followed by closing of HCN channel. That increases efficacy of synaptic imputes, neuronal activity and working memory.^23^ cAMP, cyclic adenosine monophosphate; HCN, hyperpolarization‐activated channels [Color figure can be viewed at wileyonlinelibrary.com]

Since adaptogens downregulate adenylate cyclase (*AC*) and upregulate phosphodiesterase (*PDE*) genes’ expression Panossian et al. 2013, concluded that adaptogens decrease the level of the cyclic adenosine monophosphate (cAMP) in brain cells.^23^ The authors suggested that cAMP‐mediated prefrontal cortex signaling model^542^, ^543^, ^544^ provides one possible explanation of CNS stimulating activity of adaptogens.^23^ Working memory is preserved by recurrent excitation as well as by functional interaction of G‐proteins coupled α2‐adrenoreceptors with colocalized in dendritic spines hyperpolarization‐activated channels (HCN) of prefrontal cortical neurons, Figure 12. This interaction is mediated via endogenous cAMP, which at high level promotes opening, while at low level, induces blockage of HCN channels. HCN channels opening shunts synaptic transmission onto dendritic spines, decreases cognitive performance in animals performing working memory tasks and thus improving quality of arousal and working memory, which is essential for abstract thinking, planning, and executive functions.^542^, ^543^, ^544^

Stimulation of α 2A‐adrenoreceptor decreases cAMP level and blockade of HCN channels that enhances the working memory in behavioral studies. Overall, cAMP inhibition strengthens connectivity of prefrontal cortex neuronal networks, while excessive cAMP has negative effect on the network strength.^542^, ^543^, ^544^ Since adaptogens downregulate AC and upregulate PDE that decreases cAMP level in brain cells (Figure 3), it was hypothesized that beneficial effects of adaptogens on stress‐induced and aging‐related weakening of cognitive functions in some extend is associated with their effect on cAMP–HCN mediated signaling pathway,^23^ which is increasing in stress.^542^, ^543^, ^544^ This hypothesis is consistent with studies in which adaptogens improved cognitive function in humans.^27^

Overproduction of ROS and their inadequate elimination by the innate antioxidant system in aging relate directly to transcription factors that control the expression of genes associated with cell proliferation, inflammation, and ROS production. As an example, age‐related changes in the expression of AP‐1, NF‐kB, FoxO, and Nrf2 transcription factor‐mediated signaling pathways in vascular smooth muscle cells lead to the progression of inflammaging (i.e., aging‐related low‐grade chronic inflammation) and atherosclerosis.^502^

Aging is associated with the activation of AP‐1, NF‐kB, and Nrf2 and inhibition of FoxO transcription factor‐mediated intracellular signaling pathways.^502^ Translocation of NF‐kB into the nucleus triggers the expression of multiple genes involved in inflammation.^502^

- Adaptogens downregulate NF‐kB translocation and expression, NF‐kB signaling, and NF‐kB mediated inflammation^326^, ^336^, ^434^, ^436^, ^437^, ^441^, ^545^, ^546^, ^547^, ^548^, ^549^, ^550^, ^551^, ^552^, ^553^, ^554^, ^555^, ^556^, ^557^, ^558^, ^559^, ^560^;
- adaptogens downregulate Fos and Jnk, the components of AP‐1 transcription factor (Table 9), which regulates many genes involved in cell proliferation, migration, ROS production, and extracellular matrix degradation^17^, ^502^, ^561^;
- adaptogens switch FoxO‐dependent responses from apoptosis promotion to stress‐resistance in response to oxidative stress^95^; and
- adaptogens activate Nrf2 signaling. Normally, Nrf2 is located in bound form in the cytosol with reduced kelch‐like ECH‐associated protein 1 (Keap1). Dissociated Nrf2 translocates to the nucleus, where it triggers the transcription of phase II or other adaptive response genes, including enzymes involved in GSH metabolism, NQO1,2, and HO‐1.^226^, ^550^, ^553^, ^554^, ^558^, ^561^, ^562^, ^563^, ^564^, ^565^, ^566^, ^567^, ^568^, ^569^, ^570^, ^571^, ^572^, ^573^, ^574^, ^575^, ^576^, ^577^

The efficacy of adaptogens in the treatment of acute respiratory tract diseases is possibly also partially associated with the downregulation of proinflammatory NF‐kB signaling in various cells and tissues involved in the acute inflammatory response.

*In conclusion*, the observed beneficial effects of adaptogens in aging‐related disorders (Table 10) include neurodegeneration, atherosclerosis, and impaired apoptosis.^23^, ^368^, ^369^

**Table 10:** Aging‐associated diseases and genes involved in pathogenesis and progression, up‐ or downregulated by adaptogens tested in isolated neuroglia cells

Cancer and gastrointestinal	Adenocarcinoma, prostate carcinoma, colon and colorectal cancer, cancer, gastrointestinal tumor, gastric mucosa atrophy	132 genes
Organismal injury and abnormalities	Physical disability	*PDE11A, PDE3A, PDE4D*upregulated
	Degeneration of retinal cone cells—inhibition	*AIPL1*downregulated, *CNGB3*upregulated
	Atrophy of gastric mucosa	CCKBRdownregulated
	Hypoestrogenism	*ESR1*downregulated
	Postmenopausal vulvar atrophy	*ESR1б MTNR1A*downregulated
	Pain—inhibition	*KCNK10, PDE11A, PDE3A, PDE4D, SCN2B*upregulated
	Cone dystrophy	*CDHR1, ESR1*downregulated
	Pelvic organ prolapses	*CNGB3, SERPINA1*upregulated
Dermatological	Rosacea	*AKR1D1*upregulated, *MMP8*downregulated
Inflammatory and pulmonary	Pulmonary emphysema‐ inhibition	*PDE11A, PDE3A, PDE4D, SERPINA1*upregulated
	Bronchiectasis	*PDE11A, PDE3A, PDE4D*upregulated
	Chronic bronchitis	*MMP8, MTNR1A*downregulated
Neurological and psychological	Non‐24‐h sleep‐wake disorder	*MTNR1A*downregulated
	Sleep‐wake schedule disorder	*PDE3A*upregulated
Urological	Recurrent urinary tract infection	*ESR1*downregulated
Cardiovascular	Ischemic cardiomyopathy	*PDE11A, PDE3A, PDE4D, PPP1R1A*upregulated
	Cholesteryl ester transfer protein deficiency	*CETP*downregulated
	Angina pectoris	*PDE11A, PDE3A, PDE4D*upregulated
	Cerebral small vessel disease	*PDE3A*unregulated
Skeletal and connective tissue	Osteochondrodysplasia	*COL9A1*downregulated, *PDE4D*upregulated
Metabolic	Estrogen resistance	*ESR1*downregulated

#### 5.1.5 Adaptogens in regulation of energy homeostasis

**Figure 13:**
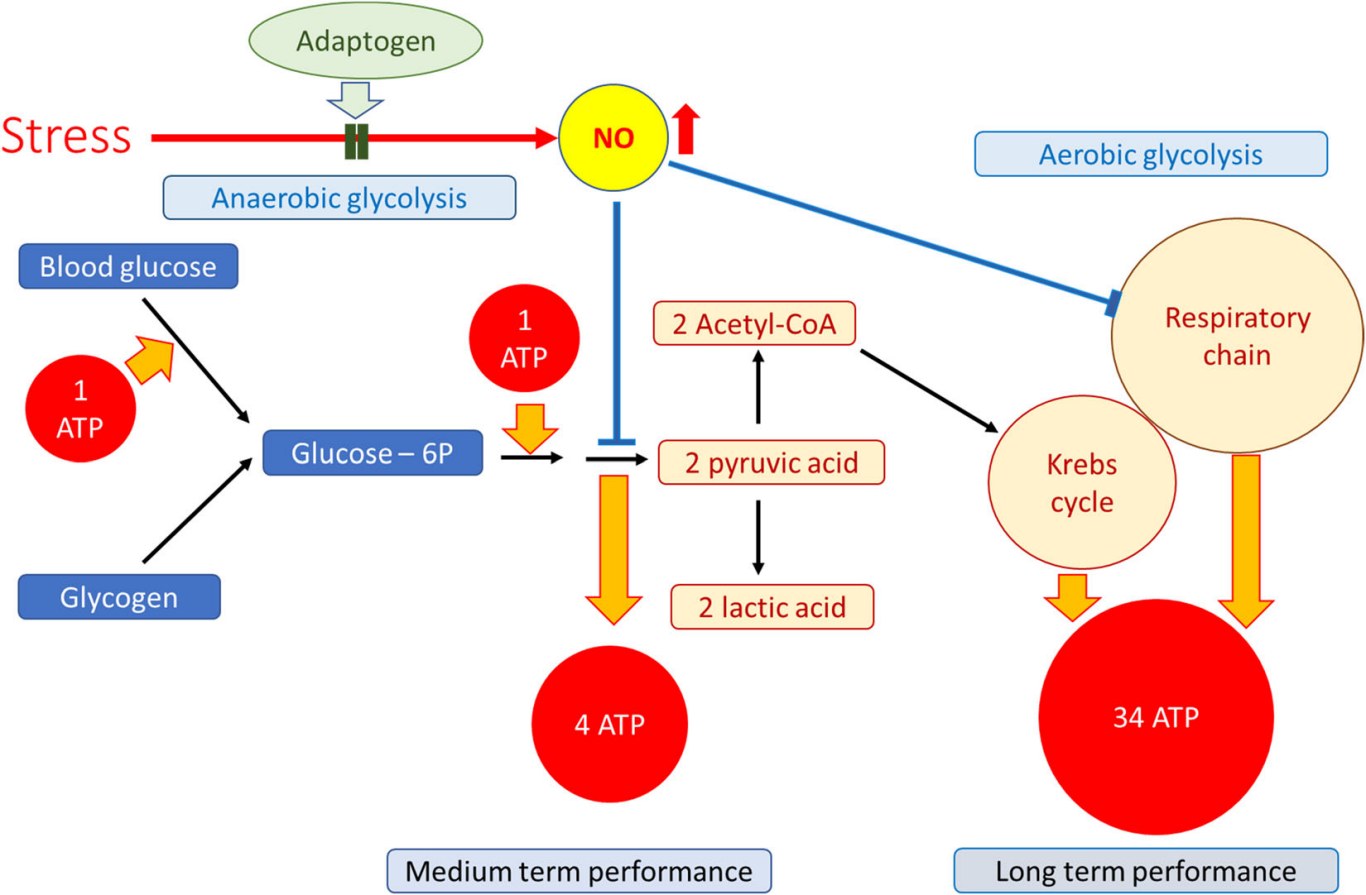
Schematic representation of the potential molecular mechanism by which generation of nitric oxide (NO) strongly inhibits the production of cellular energy through two mechanisms: inhibition of mitochondrial respiration by reversible (from constitutive isoforms of nitric oxide synthase [NOS]) and irreversible (from inducible NOS [iNOS]) inhibition of cytochrome P450^578^; and glycolysis inhibition through modification of the SH‐groups of glyceraldehyde 3‐phosphate dehydrogenase.^579^ In anaerobic glycolysis, muscle glycogen is converted to lactic acid via glucose‐6‐phosphate, yielding three ATP molecules for each glucose residue. Aerobic oxidation of glucose is required for the sustained exercise and provides 34 ATP molecules per glucose residue via the Krebs cycle and the respiratory chain, a process that occurs 1 min after anaerobic ATP generation. If a sufficient supply of ATP is not generated, anaerobic glycolysis is continued. NO levels increase during stress, consequently decreasing performance by inhibiting ATP production. Adaptogens prevent stress‐induced increases in NO,^18^ and as such, ATP production remains efficient and performance and endurance are increased.^26^ Updated from authors’ drawings^26^ [Color figure can be viewed at wileyonlinelibrary.com]

Adaptogens prevent stress‐induced increases in NO, and as such, ATP production remains efficient and performance and endurance are increased.^18^ Putative mechanisms of ATP generation are shown in Figure 13.

In addition to this possible source of ATP generation stimulated by adaptogens, apparently there are some other mechanisms of regulation of energy homeostasis by adaptogens. Thus, adaptogens presumably decrease c‐AMP level in brain cells by deregulation the expression of genes involved in regulation of cAMP.^23^ Consequently, low levels of c‐AMP decreases protein kinase A (PKA) overall activity. PKA activation effects vary with the cell type; for example, PKA stimulates lipases in adipocytes, while in myocytes and hepatocytes PKA increases glucose formation and its catabolic transformation to pyruvate (glycolysis). This provides free energy in the form of adenosyl triphosphate (ATP) and nicotine adenine dinucleotide, reduced (NADH), which are important in stress response. The regulation of cAMP levels and PKA activity is one of mechanisms of regulation of energy homeostasis, somewhat a metabolic switch between catabolism and anabolism. Stress‐induced catabolic transformations are induced by downregulation of cAMP and PKA by adaptogens. Apparently, PKA is involved in “energy‐saving” effect of adaptogens that favors ATP‐consuming anabolic pathways. Increased intracellular ATP levels and prevention of ATP‐conversion (to cAMP) is due to an inhibition of adenylate cyclase by adaptogens. An increased storage of ATP seems to represent an energy source for other ATP‐dependent metabolic conversions. This is consistent with the concept of ATP generation induced by adaptogens and their potential benefits in aging‐related diseases and fatigue.^23^

### 5.2 Synergy and antagonism of several plants as background for the discovery of new drugs with better efficacy and safety

Kampo tradition uses fixed combinations of medicinal plants in standardized proportions. The idea to combine two or more plants or substances, which will be stronger than any ingredient alone, is very attractive for several reasons: the ingredients may have different targets and mechanisms of action in human organisms, and therefore better effect as a combination; and the combination can be used at lower doses and may be less toxic if any ingredient contains a toxic impurity. The ingredients can also act synergistically, thereby providing new unique effects that are not possible to obtain by any ingredient independently. Synergistically means that the combination is active, while the ingredients separately are inactive.^17^, ^23^, ^25^, ^451^ Synergy can be also interpreted as the generation of new pharmacological activity, which is only specific for the combination of two or more ingredients.^17^, ^23^, ^451^, ^580^ This is a fantastic phenomenon that has yet to be fully understood; however, it is has been observed in various interactions at different intracellular, extracellular, organism, social, and other levels of communications.

This comparison is in line with our observations made during our analysis of the gene expression transcriptome‐wide microarray profiles of isolated neuroglia cells after incubation with several adaptogenic plant extracts, their combinations, and purified compounds.^17^, ^23^, ^25^, ^451^ It was concluded this experimental model is very useful for assessing synergistic and antagonistic interactions of various plant extracts, with the aim to discover an unexpected pharmacological activity of new combinations or to rid of adverse effects of ingredients due to their interactions within intracellular molecular networks.^17^, ^25^ Further downstream effect analysis of mRNA microarray data enables prediction of pharmacological effects of fixed combinations.^17^, ^25^, ^451^

For example, it was found that the fixed combination of *Eleutherococcus* and *Andrographis* might be useful for the treatment of encephalitis because of the synergistic inhibition of the expression of a number of genes of the molecular network involved in the development of encephalitis, whereas neither *Eleutherococcus* nor *Andrographis* individually has an effect on these genes.^25^ Although microarray analysis did not provide the final proof of the efficacy of this fixed combination in humans with encephalitis after its oral administration, it provided information regarding its predictable (*z*‐score > 2) effects on diseases and biological functions as well as insights into putative genes and directions for future research and possible implementation into practice.

This approach was implemented for the assessment of the synergistic and antagonistic interactions of *R. rosea* (RR), *W. somnifera* (WS), *E. senticosus* (ES), *Rhaponticum carthamoides* (RC), *Bryonia alba* (BA), and melatonin (M), with the purpose of predicting the potential pharmacological and toxicological profiles of their combinations (RR–BA, RR–WS, WS–M, RC–ES–WS).^17^, ^25^, ^451^

It was found that WS in combination with melatonin synergistically deregulates several genes involved in the regulation of glucagon, the main catabolic hormone that increases the concentration of glucose and fat in the bloodstream, suggesting that WS–M might be useful for the prevention of type 2 diabetes.^17^ Another synergistic interaction of WS with RR induced the deregulation of 20 genes, 10 of which contribute to the predicted activation of neuronal development, suggesting a beneficial effect of this combination on age‐related decline in memory and cognitive functions.^17^

These models take into account interactions within the biological network, which are important if medications act at various targets in the network or if homeostatic feedback mechanisms are effective. System pharmacology models are useful to describe synergistic mechanisms of action of complex combinations of medicinal plants. The term synergy is appropriate for interactions of two or more ingredients leading to qualitatively new pharmacological effects, *e.g*., to the expression of genes that cannot be obtained by any single ingredient independently. On the contrary, antagonism occurs as a result of the interaction of several ingredients in a combination, which leads to the absence, reduction or prevention of the effects of any individual ingredient in this combination.^17^, ^23^, ^25^, ^452^

## 6 CHALLENGES AND REGULATORY ISSUES

### 6.1 Terminology

The term *adaptogens*, like the terms *antioxidants* and *vitamins*, is not yet commonly used to refer to a distinct pharmacological group despite the fact that the terms *adaptogenic activity* and *adaptogen* have been adopted by drug regulatory authorities and general practitioners in Europe, the United States, and Asia.

In 2008, the European Medicines Agency published “Reflection Paper on the Adaptogenic Concept,” which was based on and which refers to the 18 review articles published in 1947–2005 mainly including studies on *Eleutherococcus* and a few other adaptogens.^581^ It this review, HMPC (anonymous authors) concluded:

- *The principle of an adaptogenic action needs further clarification and studies in the preclinical and clinical area. As such, the term is not accepted in pharmacological and clinical terminology that is commonly used in the EU*.
- *The HMPC is aware of the fact that numerous preclinical and clinical studies have been performed with the view to proving the concept of an adaptogen. However, the clinical data have a number of shortcomings such as deficiencies in the description of inclusion and exclusion criteria, description of the medication, diagnosis, study design, analysis, etc. There is a wide range of clinical conditions that have been investigated and in some studies the number of patients was very small. None of the studies would be sufficient to substantiate efficacy of Eleutherococcus preparations in a clearly defined clinical condition, although, in total, the data available are sufficient to justify further research into the concept of adaptogens*.
- *As the term “adaptogen” is considered not appropriate for a marketing authorization, more clinical studies and data on the efficacy in a well‐defined clinical condition would be necessary*.
- ***The concept of adaptogens is sufficient to be considered in the assessment of traditional herbal medicinal products*** (*e.g., monograph on Eleutherococcus root*).^3^

Since the publication of “Reflection Paper on the Adaptogenic Concept,” an enormous number of studies that significantly enrich the current knowledge of the pharmacology, clinical efficacy, and mechanisms of action of adaptogens have been published. The term *adaptogens* has been used in numerous publications indexed in PubMed that clearly show that the statement “as such, the term is not accepted in pharmacological and clinical terminology that is commonly used in the EU” is now far from reality.

Various adaptogenic herbal medicinal products, which are formally divided into two categories (EC–traditional, used for at least 30 years, including at least 15 years within the EU; and well‐established use, used within the EU for at least 10 years, with recognized efficacy and an acceptable level of safety), have a commonly acceptable level of safety and efficacy in various diseases. Nevertheless, more evidence from large‐scale, well‐controlled clinical trials of high‐quality uniform botanicals and their phase III pharmacovigilance data are essential for further implementation in common practice, at least for decreasing the risk of disease progression and as adjuvant therapy in infections and chronic diseases.

In other assessment reports,^1^, ^2^ the HMPC concluded that the preparations of three adaptogenic plants can be officially used as traditional herbal medicinal products.

- Rhodiola: for temporary relief of symptoms of stress, such as fatigue and sensation of weakness, in Austria, Italy, the Netherlands, Spain, Sweden, and the United Kingdom.^1^
- Ginseng^2^ and Eleutherococcus^3^: for the relief of symptoms of asthenia (abnormal loss of strength and energy) such as tiredness and weakness in France, Germany, Lithuania, Poland, Spain, and Sweden.^2^, ^3^

Furthermore, several *Eleutherococcus* products have marketing authorization in Germany and Denmark as herbal medicinal products with well‐established use as tonics for invigoration in individuals with fatigue and impairment, in decreasing capability and power of concentration as well as against tiredness, and in periods of convalescence.^3^

Similarly, a large number of ginseng products have marketing authorization in Austria, Belgium, Denmark, France, Germany, Ireland, Latvia, Poland, Portugal, and Spain as herbal medicinal products indicated for use “as a tonic in case of tiredness and weakness and decreased mental and physical capacity as well as in concentration,” in “asthenia, such as lack of concentration, fatigue, weakness, tiredness, lack of vitality or in convalescence,” and in “exhaustion fatigue and at convalescence; can be tried in lack of concentration in middle‐aged and elderly when other causes to the condition have been excluded.”^2^

### 6.2 Consistency of the results of clinical studies

The most important challenge is related to the evidence of the clinical efficacy of adaptogens for the treatment of many stress‐induced and aging‐related diseases, which should be demonstrated in large‐scale randomized, double‐blind, comparator‐controlled unbiased clinical studies.

Studies on well‐defined preparations often show contradictory results, which is a common trait of herbal preparations per se. Although numerous studies of adaptogens have suggested an advantageous safety and tolerability profile as compared with conventional drugs, we also acknowledge several disadvantages of herbal preparations per se. Herbal preparations, although standardized to active constituents, are still very complex mixtures of many compounds and may have variable positive and negative effects depending on factors that have a crucial impact on the reproducibility of pharmacological activity (e.g., growing conditions, regional territory, and genus differences). Difficulties in manufacturing place herbal preparations at a disadvantage against conventional drugs, which are single compounds that remain identical and reproducible from batch to batch during production. In addition, various differently standardized herbal preparations of the same medicinal plant may have different pharmacokinetic and pharmacodynamic dose–effect responses. For example, the maximal active antifatigue, antidepressant, and antistressor dose of the SHR‐5 brand of *R. rosea* extract^355^, ^356^, ^357^, ^360^ might be inactive for a different extract of *R. rosea* despite the fact that both products are extracted from *Rhodiola* roots that have different chemical compositions.^350^, ^582^ Finally, we acknowledge the difficulties in producing herbal medicinal products that provide reproducible effectiveness over time, and this represents a serious challenge and limitation of herbal medicinal products and dietary supplements in general. However, despite these limitations, the development of herbal preparation for the prevention and treatment of many diseases is of great interest and has promising potential for safe, effective, and affordable therapies with superior tolerability and a low incidence of adverse events. More evidence from properly controlled clinical studies is required to support health claims and indications of pharmaceutical‐grade herbal preparations for use in disease treatment.

### 6.3 Network pharmacology and systems biology models for assessment of pleotropic activity of adaptogens

Adaptogens are the multitaskers in terms of their pharmacological effects.

Adaptogens have pharmacologically pleiotropic effects, including antistress/antifatigue, stimulating, tonic, antidepressant, neuroprotective, cardioprotective, hepatoprotective, gastroprotective, antioxidant, autoinflammatory, immunomodulatory, antitumor, antiviral, antibacterial, and hypoglycemic activity.^1^, ^2^, ^3^, ^4^ This polyvalent activity is due to their action on genes encoding hormones, transcription factors, and other regulatory proteins, which play a key role in the regulation of many canonical intracellular signaling pathways and molecular networks as well as extracellular communication in the neuroendocrine–immune system.

The specificity of the pharmacological action of various adaptogenic plants depends on both the chemical compositions of the extracts and the dose. Product‐specific (or compound‐specific) activity is theoretically possible to achieve in the smallest dose/concentration, when a compound selectively interacts with only one receptor type, which can trigger minimal signaling pathways in a molecular network. Although the effector (ligand) molecule at higher doses can nonspecifically interact with numerous molecules of several networks, this may cause both feedback downregulations and antagonistic interactions of various molecular networks, resulting in quite different pharmacological responses and toxic effects.

At low and normal doses, adaptogens act as mild stress mimetics, increasing the homeostatic range (Figure 1) and resulting in increased resistance to stress. At higher doses, they may suppress inflammation and therefore prevent premature aging and maintain health and vitality. This is the “specific” difference of adaptogens, which activate adaptive signaling pathways and increase survival and resiliency from stress, from some other natural compounds, the so‐called PAINS (PAn‐assay Interference compouNdS), such as toxoflavin, epigallocatechin gallate, genistein, and resveratrol. Quercetin, β‐sitosterol, rutin, and curcumin do not comply with these criteria, despite nonspecific pleiotropic effects in numerous in vitro experiments.^583^

Adaptogens stimulate neurogenesis and exhibit neuroprotective activity, suggesting their potential benefits in neurodegenerative disorders. Surprisingly, they trigger apoptotic signaling pathways associated with antitumor activity. Regulation of both stress‐resistance and proapoptotic genes is not necessarily a paradox. Adaptogens stimulate mediators of the stress response and transcription factors,^17^ which may orchestrate different patterns of gene expression based on the dose of adaptogens, perhaps activating stress‐resistance genes normal or small doses, but proapoptotic genes at high doses beyond a certain threshold. Possibly, adaptogens regulate different genes in different cell types, causing apoptosis in some cells (*e.g*., cancer cells) while promoting survival in others (e.g., in neurons and glia cells). Importantly, the induction of apoptosis by adaptogens may cause the death of damaged or abnormal cells, which may extend the lifespan of the entire organism.

The adaptogenic process is can be studied very well using “systems biology” and “network pharmacology” approaches, which has the potential to provide plant‐based treatments for complex diseases, chronic conditions, and syndromes. This is a remarkably complex system of synergistic interactions of molecular networks and cellular communication systems that quite literally add up to more than the sum of the parts. It also requires a detailed understanding of disease concepts, as we have outlined in this MS and second, the use of suitable pharmacological models to understand such effects. There can be no one to one correlation between use as an adaptogens and a specific model, and the suitability of a model needs to be assessed carefully before starting experimental approaches. Such approaches can help understanding these complex systems better and this is a key challenge in the future.

### 6.4 Safety and pharmacokinetic of adaptogens

The published literature on *Rhodiola*, *Eleutherococcus*, *Withania*, ginseng, and *Schisandra* does not provide reasons for safety concerns, and herbal preparations containing adaptogens are not harmful when prepared and used in specified conditions. No serious adverse events have been reported from clinical trials, epidemiological studies, or pharmacovigilance reporting that can be clearly correlated with the ingestion of adaptogens (EMA assessment reports 2012–2014).^1^, ^2^, ^3^, ^4^ It might be suggested that such a high tolerance in humans and a low rate of adverse events might be due to their poor absorption and low bioavailability. However, the data available on absorption, distribution, metabolism, and excretion of the active constituents of adaptogenic plants show that some of these have high bioavailability and are quickly absorbed and widely distributed in all organs and tissues involved in the regulation of the neuroendocrine–immune system. Others are metabolized into more active metabolites or significantly affect gut microbiota, which plays an important role in the maintenance of homeostasis and the development of several chronic diseases, including colitis‐associated colorectal cancer, among others.

Thus, the earliest pharmacokinetic studies of adaptogens^584^, ^585^, ^586^ that intraperitoneally administered 3H‐labeled eleutheroside B (with radioactivity localized in the aglycone, 52 µCi/mmol) in rats demonstrated that eleutheroside B is quickly absorbed into the blood and distributed in the liver, kidneys, adrenals, pancreas, thymus, spleen, heart, testes, brain, and hypophysis. The extent to which a compound is distributed throughout the body has a large impact on its therapeutic utility.

The highest concentration of the label in the blood was observed 15 min after administration of the 3H‐labeled eleutheroside B, and this concentration dropped sharply within 4 h and was eliminated mainly through the renal system with urine. Approximately 35% of the labeled drug was eliminated via the urine 2 h after administration, 55% after 4 h, and 90% after 48 h. A small amount of radioactivity (2.5%–3%) was eliminated with the feces. Most of the labeled drug accumulated rapidly in the organs and tissues. In fact, after only 15 min, 88% was absorbed and retained at a high level for a rather long duration. After 8 h, up to 30% of the administered drug was still retained in the organs and tissues. This represents an exceptionally high level of incorporation of labeled eleutheroside B into the liver and kidneys with subsequent rapid removal from these organs. The high levels of labeled eleutheroside B in the pancreas can probably be attributed to its active participation in the digestion process and to its synthesis of two important hormones: insulin and glucagon. The accumulation of eleutheroside B in the adrenals for up to 4 h suggests its influence on the hypophysis–adrenal cortical system. In the brain, a minimal level of incorporation of the radioactive label with an insignificant reduction over time was observed. Eleutheroside B does not pass the blood–brain barrier.^584^, ^585^, ^586^ Interestingly, the bioavailability of individual eleutherosides B and E after oral administration of an aqueous extract of *E. senticosus* was significantly increased as compared with the oral administration of single compounds. Both eleutherosides are metabolized and excreted primarily from the liver and kidney.^587^ The absorption of orally administered eleutherosides^588^ and isofraxidin^589^ was also rapid, with the maximum concentration noted at 0.4 and 0.2 h, respectively.

Lipophilic compounds such as lignans are well distributed in the tissues and organs, where their content is higher than that in blood plasma.^590^

The absorption of any potential therapeutic is a critical consideration, especially for oral dosing. Many pharmacokinetic studies of other adaptogens, including clinical trials, have been performed.^339^, ^584^, ^585^, ^586^, ^587^, ^588^, ^589^, ^590^, ^591^, ^592^, ^593^, ^594^, ^595^, ^596^, ^597^, ^598^, ^599^, ^600^, ^601^, ^602^, ^603^, ^604^, ^605^, ^606^, ^607^, ^608^, ^609^, ^610^, ^611^ Some of these studies provided evidence of the level and steady‐state concentration of active compounds in the blood of human subjects who received oral administration of the herbal drugs in therapeutic doses. These concentrations were in line with those used in in vitro studies.^591^, ^598^ As an example, the concentration of andrographolide (the active compound of *Andrographis* and its combination with *Eleutherococcus*) in blood plasma of human subjects was approximately 3.5 µM at 2 h after drug uptake,^591^ which is adequate for exhibiting an anti‐PAF effect in vitro (EC50, 5 µM).^440^ A comparison of the results obtained in humans and rats showed that the pharmacokinetics of andrographolide are similar in both species. It was found that andrographolide is rapidly and almost totally absorbed (T1/2abs of about 25 min) into the blood (bioavailability = 91%, *F* = 0.91) after oral administration of Andrographis extract at a therapeutic dose (20 mg/kg). Thus, in the absorption phase, the concentration of andrographolide in the blood is not significantly changed during the first 1.5 h and increases to a maximal level 2 h after oral administration. It binds intensively with blood proteins and is redistributed between blood and tissues within 1 to 2 h. The elimination half‐time is in the range of 2–7 h.^591^ The tissue distribution study of andrographolide revealed the highest tissue concentration in kidney, followed by the liver, spleen, and brain, whereas an almost identical concentration was observed in the heart and lungs.^592^

The pharmacokinetics of three active compounds (tyrosol, rhodioloside, and rosavin) of *R. rosea* extracts were studied in rodents^597^, ^598^, ^599^, ^600^, ^601^, ^602^, ^603^ and healthy volunteers.^597^ Salidroside was quickly absorbed into the blood of rats (*t*_max_ = 1 h; bioavailability: 75%–90%) and metabolized to tyrosol within 2 h after oral administration of the *R. rosea* extract. The concentration of tyrosol attained its maximum value within 1.5–2.0 h and then decreased exponentially to basal level within 3 h after oral administration of the extract. Many of the measured pharmacokinetic parameters of purified salidroside were significantly different when the pure compound was administered (*C*_max_, *V*_dis_, AUC, *t*‐1/2, and higher *t*_max_ and CI) rather than the plant extract. Rosavin had a lower bioavailability (20%–26%) and was eliminated from the blood within 2 h. The pharmacokinetics of rosavin in humans are different from that in rats. For example, both *t*_max_ (2 h) and elimination rate were longer in humans after oral administration of *Rhodiola* tablets in a therapeutic daily dose. The maximal concentration and elimination half‐life of salidroside were two‐ to threefold higher than those of rosavin. The elimination of salidroside from the blood was 1.8‐fold longer than the elimination time of rosavin. The beneficial effect of *R. rosea* on mental performance in humans, which was observed 1 h after oral administration and lasted for more than 3 h, is worth noting. During this time period, the concentration of salidroside in human blood was about 587 ng/ml after 1 h and 483 ng/ml after 4 h.^597^ It was found that after intravenous administration, salidroside was extensively metabolized to tyrosol and then distributed to various organs and cleared rapidly. The highest levels of p‐tyrosol were detected in the heart, followed by the spleen, kidney, liver, and lungs.^603^

It should be noted that many of the measured pharmacokinetic parameters of purified salidroside were significantly different when the pure compound was administered (*C*_max_, Vdis, AUC, *t*‐1/2, and higher *t*_max_ and CI) rather than the total plant extract,^597^ indicating an interaction with the other constituents of the plant extract.

It should also be emphasized that the biological activity of the preparations of *R. rosea* is not entirely due to salidroside and tyrosol but rather to the entire complex of substances that are extracted from *R. rosea*. That is also true for ginseng, *Schisandra*, and presumably for all other adaptogens.

The pharmacokinetics of different active ginseng compounds have been studied in both animals and humans.^2^ The bioavailability of ginsenosides is low after oral administration, but the pharmacokinetic behavior differs among various ginsenosides.^35^, ^611^ The highly glycosylated ginsenosides Rb1, Rb2, Rc, Rd., Re, Rg1, and Rg2 have poor stability in the gastrointestinal tract, and they are easily converted into monoglycosides and aglycone ginsenosides (e.g., CK, Rh2, Rh1, and F1) by gastric acid and/or the intestinal flora.^35^, ^611^, ^612^, ^613^, ^614^, ^615^, ^616^

After oral administration, blood concentrations of ginsenosides are high, but their absorption rate is low. Both the absorption profile of ginsenosides in the intestinal mucosa and the availability of intact ginsenosides and their metabolites from the intestines are exceptionally low.^35^, ^611^ The maximal concentration of ginsenosides in plasma is reached within 2 h, suggesting that they are rapidly absorbed and distributed in tissues. Rg1, Re, Rb1, and Rc reach the brain, but their concentrations decline rapidly over time.^35^, ^617^ Rg1 and Re are more readily distributed in the brain, and they are considered the main components directly affecting the neurons of the CNS.^617^ The plasma level of ginsenosides indicates that protopanaxadiol ginsenosides have higher concentrations and longer half‐lives than protopanaxatriol ginsenosides.^35^, ^617^ After the biotransformation of ginsenosides, the microbiota in the gut produces deglycosylated products.^358^, ^618^, ^619^, ^620^ The intestinal bacteria isolated from human feces and some food‐derived microorganisms as well as fungi from soil around ginseng roots convert glycosylated ginsenosides to compound K,^612^, ^620^, ^621^ which has great potential for cancer chemoprevention.^342^ Compound K was the only ginsenoside detected in plasma and urine after the oral administration of Rb1.^622^ Deglycosylated products are better absorbed than ginsenosides based on their greater ability to permeate biological membranes.^623^

EMA assessment of the available literature suggests that ginsenoside metabolites contribute substantially to the pharmacological effects of ginseng.^2^ The metabolites are well distributed to most of the tissues.^593^ It was concluded that metabolites of ginsenosides produced by gut microbiota might be more biologically active than their precursors.^2^ The results of recent studies^624^, ^625^ are in line with this conclusion: the ginseng preparation with a higher content of rare ginsenosides was more active in its ability to prevent symptoms of stress such as fatigue, impaired memory, reduced concentration, and attention deficit related to daily work in healthy subjects^624^ as well as enhanced long‐term potentiation in rat hippocampal slices.^625^ An active ginseng metabolite may differ in distribution and clearance from its parent compound, and the parent compound and its metabolite may be bioactive by similar or different mechanisms.^342^

The results of herb–drug interaction studies of various adaptogens are contradictory.^1^, ^2^, ^3^, ^4^, ^598^, ^626^, ^627^, ^628^ Interactions with some CYP isoenzymes have been observed in vitro studies only in high concentrations of herbal extracts that are far beyond their blood levels when taken in the standard therapeutic doses and not associated with active markers.^1^, ^598^, ^626^, ^627^, ^628^ Few poorly conducted clinical studies in limited number of healthy subjects (lack of placebo, proper randomization, procedure for treatment compliance, pharmacokinetic data, sufficiently controlled consumption of CYP‐active food ingredients, etc.) do not provide strong, evidence supporting the clinical relevance of the interaction effects observed in vitro.

Overall, the pharmacokinetics of various compounds from adaptogenic plant extracts is different depending on their chemical structure, lipophilicity, water solubility, metabolic activity, concentration as well as the presence of other bioactive compounds in test samples. Adaptogens are distributed in all organs and tissues involved in the regulation neuroendocrine‐immune

system where they trigger the expression of hormones and key metabolic regulators of defense responses and cellular homeostasis. That is one of the likely explanations of the pleotropic effects of adaptogens. Finally, some adaptogens actively interact with gut microbiota that results in prevention of progression of chronic inflammatory diseases.

## 7 OVERALL CONCLUSIONS

The adaptogenic concept does not have a long history as analogues of TMS, even though adaptogenic plants have been used in TMS as rejuvenating herbs, qi tonics, rasayanas, and restoratives for centuries and are formally considered to be “traditional” by drug regulatory authorities in Europe and the United States. It is supported by an evidence‐based approach and statistical assessments of pharmacological and clinical studies of efficacy and safety of standardized herbal medicinal products as well as their mechanisms of action. The efficacy of plants used in TMS has been investigated using modern theories and methods of system biology and network pharmacology. In this review, we summarized our knowledge about common adaptogenic plants used as officinal medical preparations in USSR/Russia and in traditional Chinese medicine, Ayurveda medicine, and other TMS and alternative medical systems, and to provide a modern rationale for their use in the treatment of stress‐induced and aging‐related disorders. Overall, the basic principles of TMS are in line with those of the adaptogenic concept, which uses systems biology and network pharmacology models to understand the fundamentals of TMS such as “life vital energy”/qi (Chinese)/prana (Indian)/pneuma (Greece)/zorutyun (Armenian)/od (German)/ruah (Hebrew), and mana (Polynesian), which are related to adaptability. Yin‐yang balance can be interpreted as “homeostasis”, whereas “shanghuo”—as a state of threatened homeostasis and decreased resistance to stress, which is increased by adaptogens.

Adaptogens play key roles in defending organisms against environmental challenges including harmful bacteria, diseases carried by insects, excessive ultraviolet rays from the sun, and challenges from pollution, excess heat and cold, and hypoxia.

The key to understanding adaptogens is their role in establishing and maintaining adaptive homeostasis by building the body's natural resistance to stressors, which may be physical, chemical, biological, and psychological in nature. Adaptogens function like stress vaccines to activate the body's defence system and metabolic rate, reversing the negative physical effects of stress and restoring the body's balance and health.

- If the immune system is not functioning properly by overreacting or underreacting to challenges, adaptogens help restore the proper immune response.
- If the immune system is overly active, triggering allergies and asthma, rheumatoid arthritis or lupus, adaptogens lower the immune system's response and returns it to a normal level.
- If the immune system is underactive, leading to frequent colds, bronchitis, sinus or ear infections, and even pneumonia or causing anemia or digestive problems such as ulcers or chronic diarrhea, adaptogens can help strengthen the immune response, thereby ending the cycle of illness.
- If the brain chemistry is unbalanced, adaptogens can restore the balance, having profound effects on cognitive function, memory, and mood.

The power of adaptogens goes far beyond the immune system.

- Adaptogens can correct imbalances in cellular division cycles that cause cells to divide in an uncontrolled manner, eventually causing cancer.
- Adaptogens have a potential to prevent or postpone chronic diseases associated with aging, recognizing their uncanny ability to fix what's wrong, boost what's right, keep the body in balance, and prevent the body's functions from deteriorating.
- Adaptogenic effects like those seen in *Ginseng, Rhodiola, Eleutherpcoccus, Withania*, and *Schisandra* have been scientifically validated as being effective against chronic inflammation, atherosclerosis, neurodegenerative cognitive impairment (e.g., Alzheimer's disease and other forms of dementia), metabolic disorders, diabetes, cancer and a host of other aging‐related diseases.

Overall, in this review for the first time we compare and analyze common basic principles, concepts, and uses of adaptogenic plants using a cross‐cultural, comparative approach. We demonstrate that the concept of adaptogens provides a scientific rationale for adaptogenic plants traditionally used in stress‐induced and aging‐related diseases. In conclusion, the basic principles of TMS are in line with those of the adaptogenic concept, which uses systems biology and network pharmacology models to understand the fundamentals of TMS.

## CONFLICT OF INTERESTS

Alexander G. Panossian is self‐employed by the research and development company, Phytomed AB. He has an Independent Contractor Agreement with Europharma USA Inc. He has no shares or financial interest in any pharmaceutical company. All other authors declare no conflict of interests.

## AUTHOR CONTRIBUTIONS

Alexander G. Panossian initiated this project, planned and wrote the first and final draft of the manuscript. All other authors added specific parts, critically reviewed and edited the drafts, and approved the final version of the manuscript.
